# Memory T cells possess an innate-like function in local protection from mucosal infection

**DOI:** 10.1172/JCI162800

**Published:** 2023-05-15

**Authors:** Tanvi Arkatkar, Veronica Davé, Irene Cruz Talavera, Jessica B. Graham, Jessica L. Swarts, Sean M. Hughes, Timothy A. Bell, Pablo Hock, Joe Farrington, Ginger D. Shaw, Anna Kirby, Michael Fialkow, Meei-Li Huang, Keith R. Jerome, Martin T. Ferris, Florian Hladik, Joshua T. Schiffer, Martin Prlic, Jennifer M. Lund

**Affiliations:** 1 Vaccine and Infectious Disease Division, Fred Hutchinson Cancer Research Center, Seattle, Washington, USA.; 2 Department of Global Health and; 3 Department of Obstetrics and Gynecology, University of Washington, Seattle, Washington, USA.; 4 Department of Genetics, University of North Carolina at Chapel Hill, Chapel Hill, North Carolina, USA.; 5 Department of Laboratory Medicine and Pathology and; 6 Department of Medicine, University of Washington, Seattle, Washington, USA.; 7 Clinical Research Division, Fred Hutchinson Cancer Research Center, Seattle, Washington, USA.; 8 Department of Immunology, University of Washington, Seattle, Washington, USA.

**Keywords:** Immunology, Infectious disease, T cells

## Abstract

Mucosal infections pose a significant global health burden. Antigen-specific tissue-resident T cells are critical to maintaining barrier immunity. Previous studies in the context of systemic infection suggest that memory CD8^+^ T cells may also provide innate-like protection against antigenically unrelated pathogens independent of T cell receptor engagement. Whether bystander T cell activation is also an important defense mechanism in the mucosa is poorly understood. Here, we investigated whether innate-like memory CD8^+^ T cells could protect against a model mucosal virus infection, herpes simplex virus 2 (HSV-2). We found that immunization with an irrelevant antigen delayed disease progression from lethal HSV-2 challenge, suggesting that memory CD8^+^ T cells may mediate protection despite the lack of antigen specificity. Upon HSV-2 infection, we observed an early infiltration, rather than substantial local proliferation, of antigen-nonspecific CD8^+^ T cells, which became bystander-activated only within the infected mucosal tissue. Critically, we show that bystander-activated CD8^+^ T cells are sufficient to reduce early viral burden after HSV-2 infection. Finally, local cytokine cues within the tissue microenvironment after infection were sufficient for bystander activation of mucosal tissue memory CD8^+^ T cells from mice and humans. Altogether, our findings suggest that local bystander activation of CD8^+^ memory T cells contributes a fast and effective innate-like response to infection in mucosal tissue.

## Introduction

Maintaining a balance of protection from pathogens while minimizing pathology is a perpetual process in mucosal barrier tissues, and tissue-resident memory CD8^+^ T cells (CD8^+^ TRMs) have been shown to be critically important in this process. CD8^+^ TRMs are localized at tissue sites of prior viral replication and have been demonstrated to trigger an antiviral state within the tissue microenvironment (1, 2). CD8^+^ TRMs mediate rapid target cell killing, and further, T cell receptor–mediated (TCR-mediated) activation of CD8^+^ TRMs can protect against unrelated pathogens in an IFN-γ–dependent manner in the female genital tract (FGT) and skin, referred to as a sensing and alarm function of CD8^+^ TRMs (1, 2). Thus, these studies showed that TCR-mediated TRM reactivation initiates a broad antiviral state not limited to the primary pathogen. However, it is unclear whether this state can be elicited in the absence of cognate antigen. This is an important question to address, because a TCR dependency would indicate that the TRM alarm function may primarily serve to prevent secondary infections while responding to a previously encountered antigen. In contrast, if CD8^+^ TRMs localized in the FGT can mediate this broad-spectrum protection in the absence of TCR-mediated activation, it would indicate a much broader role for CD8^+^ TRMs in barrier immunity.

Importantly, memory T cells can be activated by inflammatory cytokines independently of cognate antigen interaction, and exert effector function in an innate-like manner (3–5). Furthermore, in bypassing TCR-dependent activation, these bystander-activated memory T cells act very early in the course of an infection or inflammatory event, before clonal expansion. Once activated, they produce effector molecules including IFN-γ and granzyme B, thereby earning the name bystander-activated cytotoxic CD8^+^ T lymphocytes (BA-CTLs). Early studies revealed the innate-like potential of memory CD8^+^ T cells by observing enhanced proliferation of memory CD8^+^ T cells upon injection with LPS, poly I:C, and type I IFNs in mice in the absence of antigen (6–8). Cytokine activation of memory CD8^+^ T cells can lead to IFN-γ or granzyme B production via cytokines such as IL-2, IL-12, IL-15, IL-18, and type I IFNs (9–13). Both the activation cues and the biological relevance of BA-CTLs appear to be context-dependent, since these cells have been described as both immunoprotective and pathogenic. For instance, in the setting of acute hepatitis A, *Leishmania major*, or *Borrelia burgdorferi* infection, wherein there is sustained inflammation or activation, BA-CTLs have been linked to pathology (4, 14–16). In contrast, the rapid effector response of memory CD8^+^ T cells can be beneficial when briefly activated in infections such as *Listeria* *monocytogenes*, influenza A virus, *Yersinia pseudotuberculosis*, murine γ-herpesvirus, and *Staphylococcus aureus* (4, 9, 10, 12, 13, 17–19). Thus, it is clear that non-antigen-specific memory T cells can participate in anti-pathogen immunity, though the impact on disease outcome appears to be infection- and possibly tissue-dependent.

The tissue microenvironment appears to play a critical role in modulating bystander activation of CD8^+^ TRMs. Memory CD8^+^ T cells in the secondary lymphoid organs fail to become bystander-activated during systemic inflammation, suggesting that a tissue-specific environment shapes bystander CD8^+^ T cell responses (19, 20). Ge et al. reported that lung TRMs do not require cognate antigen interactions in order to become bystander-activated and reduce the severity of bacterial pneumonia (17). This study revealed that lung tissue parenchyma-bound TRMs are bystander-activated and recruit neutrophils to reduce pathogen burden. Other studies within the context of localized infection in the lung and spleen report that circulating memory CD8^+^ T cells of irrelevant antigen specificity infiltrate tissues at early stages in response to infection (13, 20).

The female reproductive tract has a unique physiology and poses a barrier against sexually transmitted infections of global importance such as human immunodeficiency virus 1 (HIV-1), chlamydia, human papillomavirus (HPV), and human herpes simplex virus 2 (HSV-2). Further, epidemiological evidence suggests that infection with one pathogen substantially increases the risk of acquiring other infections, as observed in the case of HIV-1 and HSV-2 (21–23). We previously reported an increase in the number of CD8^+^ T cells in the vaginal tissue compared with the cervix in healthy women (24). The increase in the CD8^+^ T cells could reflect heightened immune surveillance at a steady state in the vaginal tissue, which is in perpetual contact with the external environment and serves as a barrier against invading pathogens. HSV-2 is an example of a local FGT infection, wherein shedding is recurrent and often frequent, yet is generally rapidly contained by a very low density of CD8^+^ TRMs (20, 25–28). Prior studies have characterized the protective role of CD8^+^ TRMs in the FGT (25–28), and yet mathematical modeling revealed that in situations wherein HSV-2–specific CD8^+^ TRMs could only exert contact-mediated killing, viral spread was not contained (29). Thus, we sought to investigate how memory T cells could participate in protection against local virus infection in an innate-like fashion in the FGT. Here, we use a mouse model of HSV-2 infection to characterize the relevance of innate-like, bystander-activated CD8^+^ T cells in FGT mucosal infection and disease progression. Based on our findings, we present a model wherein both peripheral and tissue-resident memory CD8^+^ T cells of irrelevant antigen specificity become bystander-activated in a TCR-independent manner in response to the inflammatory immune microenvironment within the infected tissue. These bystander-activated memory CD8^+^ T cells function very early during infection in an innate-like TCR-independent manner to partially ameliorate infection until a more robust antigen-specific response emerges.

## Results

### Memory CD8^+^ T cells with irrelevant antigen specificity provide partial protection from lethal HSV-2 infection.

We previously demonstrated that systemic immunization with a recombinant strain of *Listeria monocytogenes* (LM) expressing HSV glycoprotein B (gB) elicits CD8^+^ TRMs in the vaginal tract (25). Further, we found that this immunization conferred partial protection against lethal HSV-2 challenge in mice. However, antigen-specific CD8^+^ T cells can participate in pathogen protection in the FGT not only directly, through cytotoxic activity, but can also sound the alarm to recruit other immune cells to the tissue (30). Thus, we first sought to determine whether LM-gB immunization and subsequent HSV infection resulted in the recruitment of other, non-gB-specific, memory T cells to the vaginal tract. We took advantage of the LM-OVA-gB construct, which expresses ovalbumin (OVA) in addition to the HSV-derived gB epitope. Hence, LM-OVA-gB immunization allowed us to assess HSV-specific (gB-specific) and HSV-nonspecific (OVA-specific) cells. Since we hypothesized that bystander-activated CD8^+^ T cells could be early responder cells with innate-like functions after local challenge, we examined the OVA tetramer–positive (HSV-nonspecific) population within total CD8^+^ T cells in the LM-gB–immunized mice after HSV-2 infection (Supplemental Figure 1; supplemental material available online with this article; https://doi.org/10.1172/JCI162800DS1). We observed that 2%–4% of total CD8^+^ T cells were specific for OVA tetramer in the vaginal tract after HSV-2 infection (Supplemental Figure 1C). Since immunization with LM-gB elicits a stable HSV-specific TRM compartment in the vaginal tract, it was not surprising to observe 8%–15% of total CD8^+^ T cells specific to HSV-2 (Supplemental Figure 1C). However, over 70% of CD8^+^ T cells were of unknown specificity, and the presence of OVA-specific CD8^+^ T cells suggests that such memory T cells could participate in subsequent local immune responses.

**Figure 1:**
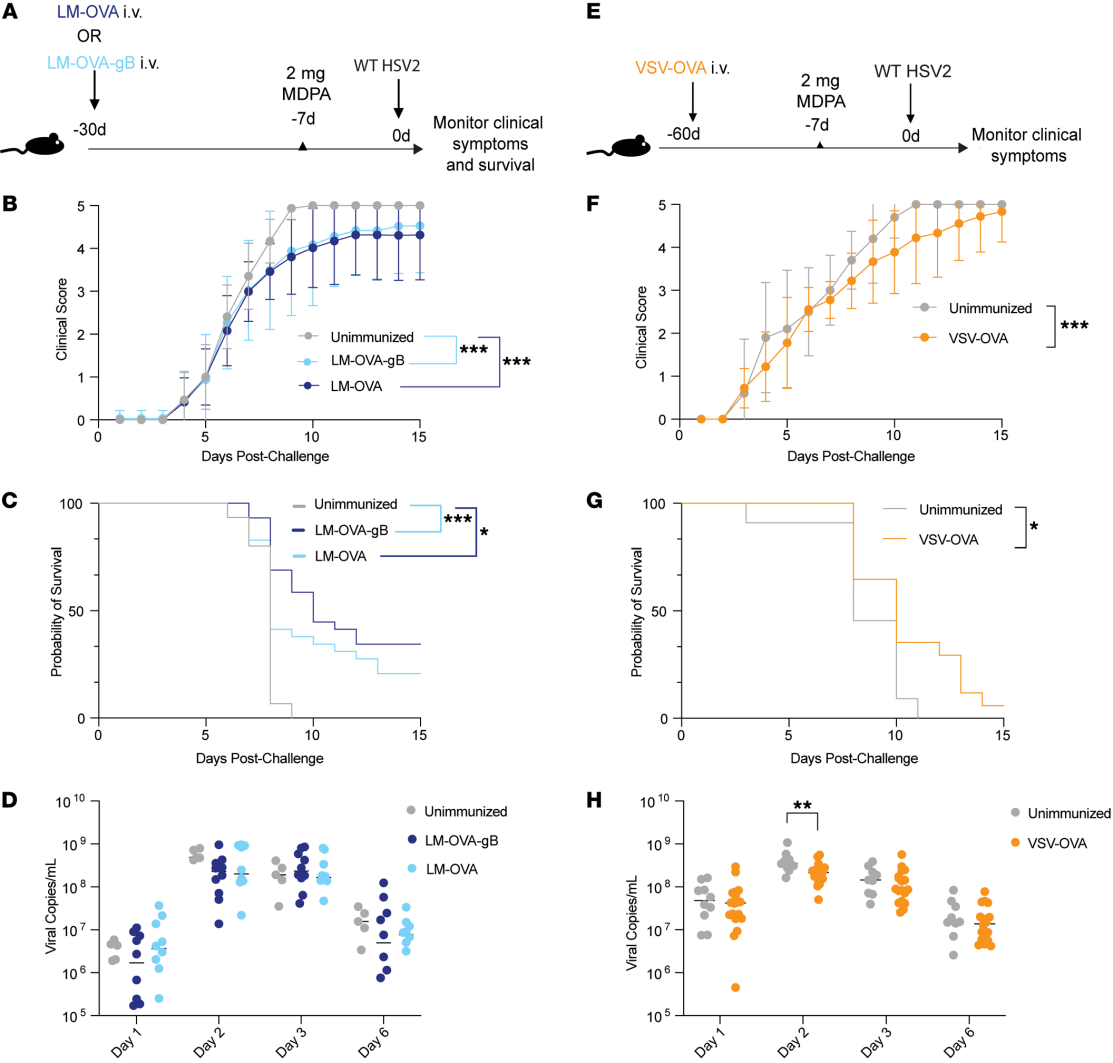
Antigen-nonspecific CD8^+^ T cells provide partial protection from genital HSV-2 infection. (**A**) Experimental schematic to compare protective efficacy of LM-OVA-gB (HSV-specific) immunization and LM-OVA (HSV-nonspecific) immunization. (**B** and **C**) Mice were monitored for 2 weeks for clinical score (**B**) and survival (**C**) after lethal HSV-2 challenge. (**D**) Vaginal swabs were collected on days 1, 2, 3, and 6 from HSV-2–infected mice that were either unimmunized, LM-OVA–immunized, or immunized with LM-OVA-gB. Viral titers were measured by RT-PCR. (**E**) Experimental schematic to assess protective efficacy of VSV-OVA immunization. (**F** and **G**) Mice were monitored for 2 weeks for clinical score (**F**) and survival (**G**) after lethal HSV-2 challenge. (**H**) Vaginal swabs were collected on days 1, 2, 3, and 6 from HSV-2–infected mice that were either unimmunized or immunized with VSV-OVA. Viral titers were measured by real time PCR (RT-PCR). For viral titers in **D** and **H**, each dot represents an individual mouse and data are pooled from 2 or 3 experiments with 10–12 mice per group. Error bars represent mean ± SD. Statistical significance was determined by 2-way ANOVA with Tukey’s multiple comparisons for **B** and **F**, by log-rank test for **C** and **G**, and by unpaired *t* test for **D** and **H**. *P < 0.05, **P < 0.01, ***P < 0.001.

After establishing the presence of the HSV-nonspecific memory CD8^+^ T cells in the vaginal tract following a local mucosal viral challenge, we wanted to establish the role of these cells in infection and disease progression. To assess whether bystander memory CD8^+^ T cells participate in limiting disease, we generated an HSV-nonspecific (OVA-specific) memory CD8^+^ T cell compartment by immunizing mice with LM-OVA. A second group of mice was immunized with LM-OVA-gB in order to generate mice with HSV-specific TRMs, as we have previously demonstrated (25). On day 30 after immunization, we intravaginally infected mice with a lethal dose of wild-type HSV-2 and monitored mice for 2 weeks for clinical symptoms (Figure 1A). Consistent with our previous study (25), we observed a significantly lower clinical score in mice immunized with LM-OVA-gB after the lethal HSV-2 challenge. Surprisingly, immunization with LM-OVA also resulted in improved clinical scores, as opposed to naive mice, which succumbed to infection within 6–8 days as indicated with a score of 5. Further, there was no significant difference in clinical scores nor survival between the LM-OVA and LM-OVA-gB immunization groups (Figure 1, B and C), suggesting a critical role for HSV-nonspecific CD8^+^ T cells in mediating protection. However, we did not observe a difference in the early mucosal viral titers in unimmunized and immunized mice (Figure 1D), suggesting that mice could be spared from early death via a neuroprotective mechanism consistent with a study by Shin et al. (26), who reported that memory CD8^+^ T cells have a limited role in controlling mucosal viral replication. Finally, to rule out the possibility of potential cross-reactive epitopes between LM and HSV-2, we adopted an approach wherein we immunized mice with OVA-expressing vesicular stomatitis virus (VSV-OVA) before HSV-2 challenge (Figure 1E). Similarly to LM-OVA immunization, we found that immunization with VSV-OVA also resulted in improved clinical outcomes and survival (Figure 1, F and G). Interestingly, we observed a reduction in the mucosal viral titers on day 2 in VSV-OVA–immunized mice compared with unimmunized mice (Figure 1H).

To validate the specific role of CD8^+^ T cells in this immune-mediated protection, we depleted CD8^+^ T cells in the LM-OVA memory mice through systemic administration of anti-CD8 depleting antibody just prior to HSV-2 infection (Supplemental Figure 2A). After confirming effective depletion of CD8^+^ T cells within the vaginal tract (Supplemental Figure 2B), we monitored disease scores after HSV-2 challenge (Supplemental Figure 2C). CD8-depleted mice immunized with LM-OVA exhibited a worsened clinical outcome and delayed survival compared with the CD8-sufficient group, thereby confirming a role for HSV-nonspecific CD8^+^ T cells in improving the disease outcome (Supplemental Figure 2D). We also observed significantly increased viral burden in the CD8-depleted mice by day 6 after infection (Supplemental Figure 2E), consistent with a critical role for CD8^+^ T cells in viral control.

Finally, we used 2 parallel approaches to validate the significance of antigen-nonspecific CD8^+^ T cells in HSV-2–mediated protection. In the first approach, we purified total CD8^+^ T cells from LM-OVA–immunized mice and adoptively transferred the cells into naive recipients infected with HSV-2 at the time of T cell transfer (Supplemental Figure 3A). Compared with the unimmunized control (10%–16%), the LM-OVA–immunized mice had a larger frequency of CD44^+^ CD8^+^ memory T cells (18%–28%), as indicated in Supplemental Figure 3B. Interestingly, we found lower clinical scores, delayed survival, and lower viral titers on day 1 after HSV-2 challenge (Supplemental Figure 3, C–E), confirming the role of antigen-nonspecific CD8^+^ T cells in alleviating disease symptoms. However, this experiment showed that the transfer of CD8^+^ T cells from unimmunized mice, which also have a preexisting memory population given that they are specific pathogen–free mice that have been exposed to some microbes and possess a population of virtual memory T cells (31, 32), is not sufficient to mediate protection. Instead, transfer of a population of CD8^+^ T cells with an enhanced frequency of memory cells elicited by LM-OVA immunization was required to reduce the disease symptoms (Supplemental Figure 3C). Using a second approach, we purified CD44-low CD8^+^ T cells (naive) and CD44-high CD8^+^ memory T cells from the LM-OVA–immunized mice to determine whether the protection by CD8^+^ memory T cells is superior to that by naive CD8^+^ T cells (Supplemental Figure 4A). We found significantly lower clinical scores in mice receiving memory T cells (Supplemental Figure 4B), though there was only a slight, non-significant difference in survival or viral titers (Supplemental Figure 4, C and D). Altogether, our data point to a role for memory CD8^+^ T cells with irrelevant antigen specificity in affording a degree of protection from lethal mucosal HSV-2 infection.

### Antigen-nonspecific memory CD8^+^ T cells are present in the vaginal tissue and show a bystander phenotype only in the infected mucosa.

**Figure 2:**
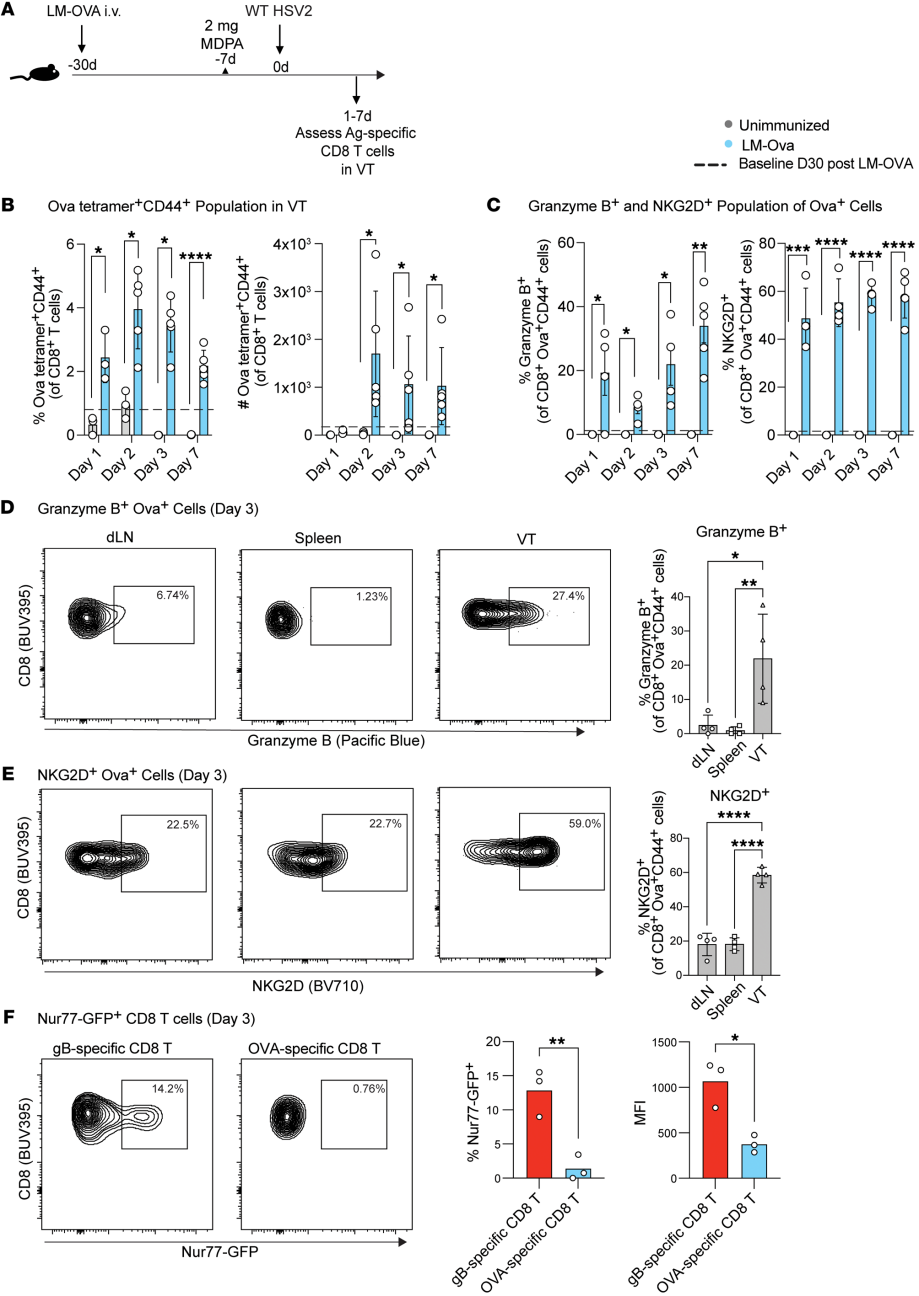
OVA-specific CD8^+^ T cells are present in the vagina after HSV-2 infection and display a bystander-activated phenotype. (**A**) Experimental schematic to generate an OVA-specific memory compartment followed by intravaginal challenge with wild-type (WT) HSV-2. Mice were euthanized on days 1–7 after WT HSV-2 challenge to assess OVA-specific CD8^+^ T cells in the vaginal tract (VT). (**B**) Frequency and counts of CD44^+^ OVA tetramer–specific cells on days 1–7 after HSV-2 challenge in LM-OVA–immunized mice. Dashed lines indicate mean of OVA-specific cells in the VT of LM-OVA–immunized mice prior to HSV-2 challenge. (**C**) Frequency of granzyme B^+^ and NKG2D^+^ population gated on OVA tetramer–positive population. (**D** and **E**) Frequency of granzyme B^+^ and NKG2D^+^ population gated on OVA tetramer–positive population within draining lymph nodes (dLN; iliac and inguinal), spleen, and VT. Each dot represents an individual mouse, and data are representative of 1–3 experiments with 4–5 mice per group. (**F**) HSV gB-specific and OVA-specific memory compartments were generated by immunization of Nur77-GFP mice with LM-OVA-gB or LM-OVA followed by intravaginal challenge with WT HSV-2. Mice were euthanized on day 3 after WT HSV-2 challenge to assess Nur77-expressing populations within gB- and OVA-specific CD8^+^ T cells in the VT. Red bars indicate HSV gB-specific population within LM-OVA-gB–immunized mice, and blue bars indicate OVA-specific population within LM-OVA–immunized mice. Data are representative of 3 experiments, each with 3 mice per group. Error bars represent mean ± SD. Statistical significance was determined by unpaired *t* test for **B**, **C**, and **F**, and 1-way ANOVA for **D** and **E**. *P < 0.05, **P < 0.01, ***P < 0.001, ****P < 0.0001.

Given our finding that non-HSV-specific memory CD8^+^ T cells provided a degree of protection from lethal mucosal infection, we next sought to characterize these CD8^+^ T cells. We hypothesized that local HSV infection after immunization with LM-OVA results in bystander activation of memory OVA-specific CD8^+^ T cells, and that this activity could underlie observed protection. Since bystander-activated CD8^+^ T cells are known to secrete cytotoxic molecules including granzyme B and express NKG2D to drive protection against unrelated pathogens (9, 33), we evaluated these markers from days 1 to 7 after infection (Figure 2, A–C). Compared with the basal OVA-specific population established at day 30 after LM-OVA immunization (indicated by dashed line), we found an increased presence of OVA-specific memory CD8^+^ T cells on days 1–7 in the vaginal tissue after HSV-2 infection (Figure 2B). We next looked at granzyme B and NKG2D expression in vaginal OVA-specific cells and observed that 20%–40% of these cells expressed granzyme B and 50%–65% expressed NKG2D on day 3 after infection (Figure 2C).

In addition to assessing mucosal OVA-specific CD8^+^ T cells, we also examined these cells in the spleen and vaginal draining lymph nodes. Since bystander activation is a localized response and is facilitated by a site-specific recruitment process of memory T cells (20), we reasoned that a local, tissue-specific environment could shape bystander responses. To explore the possibility of whether granzyme B expression within the OVA-specific population is unique to the vaginal tract in the context of HSV-2 infection, we assessed the phenotype of these cells in secondary lymphoid organs (Figure 2, D and E). Granzyme B and NKG2D expression was significantly lower in OVA-specific cells in the draining lymph nodes and spleen (Figure 2, D and E), despite the considerable number of OVA-specific cells in these tissues (Supplemental Figure 5). The unique activated phenotype of vaginal OVA-specific CD8^+^ T cells supports our hypothesis that the local tissue microenvironment resulting from local HSV-2 infection provides instructional cues, independent of cognate antigen, that lead to bystander activation within the vaginal mucosa.

Finally, we sought to demonstrate that OVA-specific CD8^+^ T cells that accumulate and become activated in the vaginal tract after HSV-2 infection are activated independently of TCR stimulus. Hence, we assessed Nur77, a marker of TCR activation (10, 34, 35), using reporter mice. Whereas HSV gB-specific vaginal CD8^+^ T cells expressed Nur77, indicating recent TCR stimulus, OVA-specific vaginal CD8^+^ T cells lacked Nur77 expression (Figure 2F). This confirms that memory CD8^+^ T cells do not rely on TCR signals to become bystander-activated in the vaginal tissue following HSV-2 infection.

### The presence and accumulation of bystander-activated memory CD8^+^ T cells in the mucosa require infiltration of circulating memory CD8^+^ T cells in a sphingosine 1-phosphate–dependent manner.

**Figure 3:**
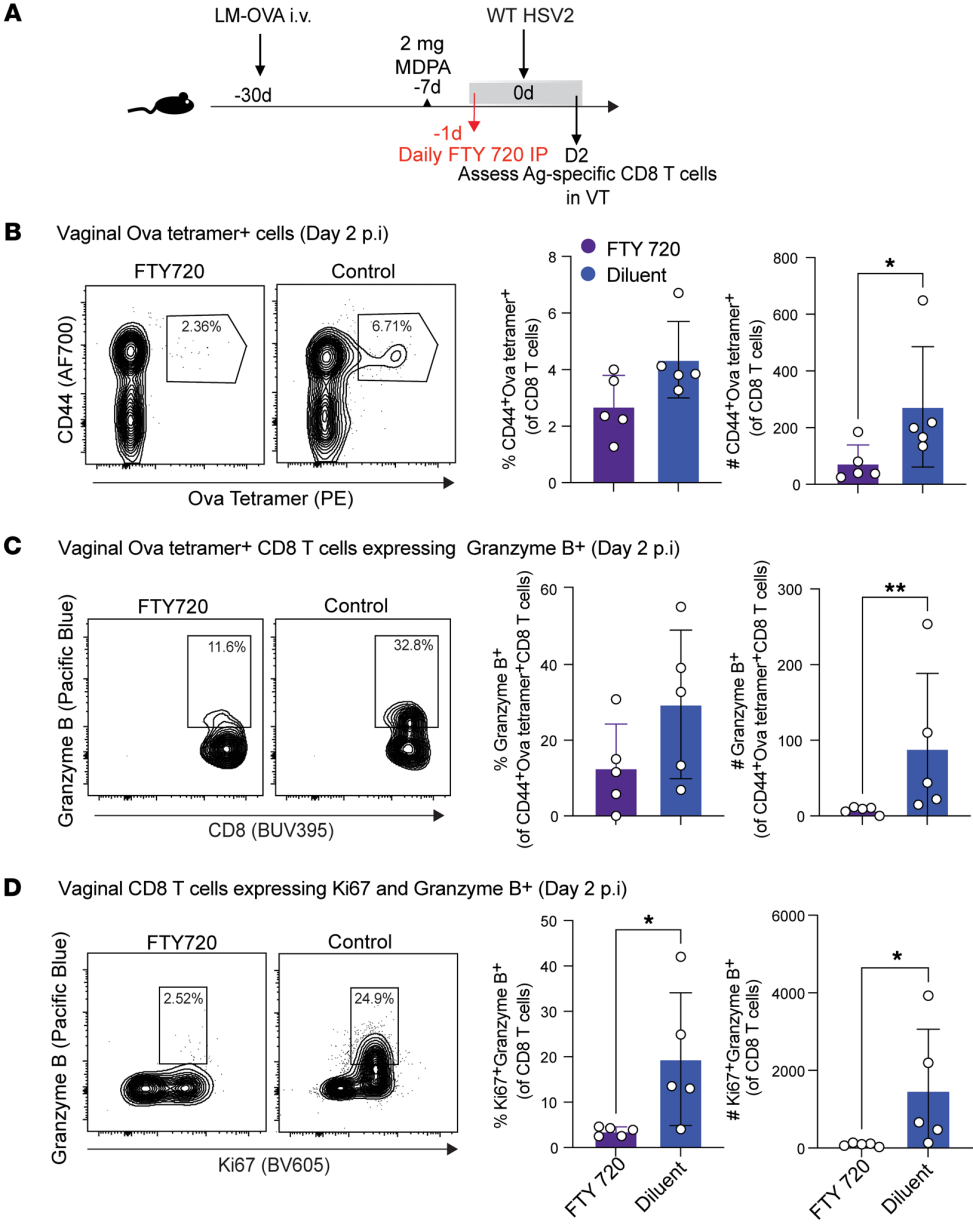
FTY720 treatment prevents accumulation of bystander-activated OVA-specific CD8^+^ T cells in the infected mucosa. (**A**) Experimental schematic to generate OVA-specific memory compartment followed by intravaginal challenge with WT HSV-2. FTY720 (1 mg/kg) or 2% cyclodextrin was administered by an intraperitoneal route on days –1, 0, 1, and 2 relative to HSV-2 infection. (**B**) Representative flow plots and graphs to assess OVA tetramer–positive subset in the VT in LM-OVA–immunized mice infected with WT HSV-2 that received either FTY720 or diluent (2% cyclodextrin). (**C**) Representative flow plots and graphs of granzyme B–expressing population within OVA^+^CD44^+^ subset. (**D**) Representative flow plots and graphs of Ki67^+^granzyme B^+^ population plotted on total CD8 gate. Each dot represents an individual mouse, and data are representative of 2 experiments with 4–5 mice per group. Error bars represent mean ± SD. Statistical significance was determined by unpaired *t* test. *P < 0.05, **P < 0.01.

Given that CD8^+^ TRMs in skin, lung, and FGT mediate broad antiviral effects against unrelated pathogens (1, 2, 19), we next sought to determine the origin of early bystander T cell accumulation in the vaginal tract. We reasoned that the vaginal bystander-activated CD8^+^ T cells we observed after HSV-2 infection (Figure 2B) could have migrated from the circulation, or they could be TRMs, established following immunization with LM-OVA, that proliferated in situ. Effector T cells rely on sphingosine 1-phosphate (S1P) receptor signaling to egress from the secondary lymphoid organs (36) and migrate to the tissue, and therefore blocking S1P signaling prevents infiltration of circulating T cells into the tissue while enabling TRM retention (37). Thus, we blocked the recruitment of T cells into the vagina during infection and observed the number and phenotype of OVA-specific bystander T cells. Starting 1 day before infection, we gave mice daily intraperitoneal injections of FTY720, an agonist of the S1P receptor, which renders cells unresponsive to the S1P receptor signals (Figure 3A). We found that the treatment with FTY720 resulted in significantly fewer OVA-specific CD8^+^ T cells in the vaginal tissue (Figure 3B), indicating that the origin of many of the OVA-specific memory CD8^+^ T cells was likely from circulation rather than solely from a pool of TRMs. Furthermore, granzyme B–expressing OVA tetramer–positive CD8^+^ T cells were also significantly reduced in numbers in mice treated with FTY720 (Figure 3C).

Finally, to explore the origin of all bystander-activated T cells, including those that are not necessarily OVA-specific, we looked at proliferating T cells based on Ki67 staining and coexpression of granzyme B within the total, polyclonal CD8^+^ T cell population and observed a significant reduction in the number of Ki67^+^ granzyme B^+^ CD8^+^ T cells after FTY720 treatment (Figure 3D). Altogether, our data demonstrate that HSV-nonspecific memory CD8^+^ T cells are rapidly recruited from the circulation to the vaginal tissue upon HSV-2 infection, where they may receive bystander activation signals to induce local proliferation and expression of cytotoxic molecules.

### Adoptive transfer of bystander-activated CD8^+^ T cells is sufficient to delay the progression of lethal HSV-2 infection and reduce early viral burden.

**Figure 4:**
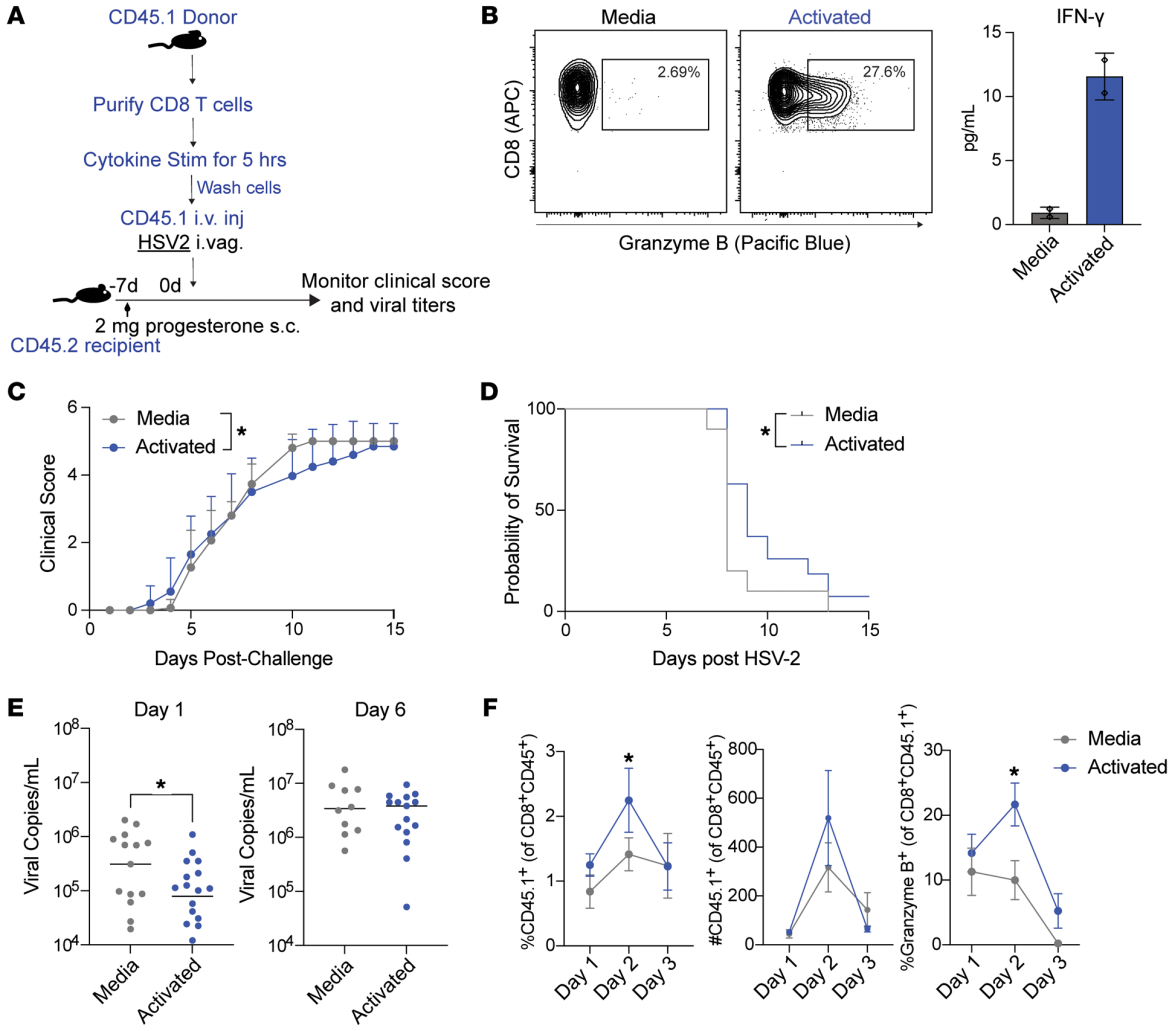
Adoptively transferred bystander-activated CD8^+^ T cells delay the progression of lethal HSV-2 infection and lower the viral burden. (**A**) Experimental outline to compare the viral burden of mice receiving CD8^+^ T cells stimulated with medium or cytokines (IL-12/15/18 plus IFN-α/β) after WT HSV-2 challenge. CD8^+^ T cells were purified from splenocytes and draining lymph nodes and were derived from CD45.1 donor mice. The cells were stimulated with medium (RP10) or cytokines (10 ng/mL IL-12/15/18 plus 1,000 U IFN-α/β; activated) for 5 hours and washed before intravenous injection into CD45.2 recipient mice. (**B**) Representative flow plots showing intracellular granzyme B staining in medium- and cytokine-treated memory CD8^+^ T cells at 5 hours. IFN-γ levels were measured by ELISA from supernatants obtained after 5 hours of incubation. (**C** and **D**) Mice were monitored for clinical score and survival after the lethal HSV-2 challenge. (**E**) Vaginal washes were obtained after HSV-2 infection, and viral titers were measured by RT-PCR on days 1 and 6 after HSV-2 infection. (**F**) Mice were euthanized on days 1–3 after WT HSV-2 challenge and adoptive transfer to assess frequency and counts of donor CD45.1^+^ CD8^+^ T cells in the VT. Percent frequency of granzyme B^+^ population was gated on CD45.1^+^ CD8^+^ T cells. Data are pooled from at least 3 independent experiments with *n* = 5–10 for the control and experimental groups. Error bars represent mean ± SEM. Statistical significance was determined by 2-way ANOVA with Tukey’s multiple comparisons and by unpaired *t* test (**E** and **F**). *P < 0.05.

Previous studies have reported that brief exposure of antigen-specific memory CD8^+^ T cells to IL-12 and IL-18 induces proliferation, increased antiviral response, and decreased viral load (5). We investigated the hypothesis that memory CD8^+^ T cells of irrelevant antigen specificity could become activated after brief cytokine exposure and could thus impact the outcome of infection. We modified an experimental methodology described by Raué et al. (5), wherein we stimulated CD8^+^ T cells from naive CD45.1 mice ex vivo with cytokines before adoptive transfer into congenic hosts that were then infected vaginally with HSV-2 (Figure 4A). We selected a cytokine combination that resulted in a robust granzyme B and IFN-γ response, and exposed CD8^+^ T cells to the cytokines for 5 hours. We confirmed that the cells stimulated ex vivo with cytokines produced granzyme B and IFN-γ intracellularly or in the culture supernatant after 5 hours (Figure 4B). After the 5-hour incubation period, the cells were washed and transferred into naive recipients intravenously, followed by intravaginal infection with wild-type HSV-2 at the time of cell transfer. Mice receiving adoptively transferred bystander-activated CD8^+^ T cells showed a significant delay in disease progression as assessed by clinical scoring and survival and exhibited significantly lower viral titers than the control mice on day 1 after infection (Figure 4, C–E), suggesting an involvement of bystander-activated CD8^+^ T cells in reducing the early viral burden. Additionally, an increased presence of the adoptively transferred CD45.1^+^ cells was detected in the vaginal tract at day 2 after CD8^+^ T cell transfer and HSV-2 infection (Figure 4F). The mice receiving activated CD8^+^ T cells also exhibited higher granzyme B expression by day 2 after infection (Figure 4F). Overall, this signified that after cytokine-mediated activation, memory CD8^+^ T cells, regardless of antigen specificity, gain enhanced cytolytic potential and play an innate-like and early role in benefiting the host by reducing viral burden upon infection.

### In vivo type I IFN treatment is sufficient to increase vaginal memory CD8^+^ T cell abundance and bystander activation.

**Figure 5:**
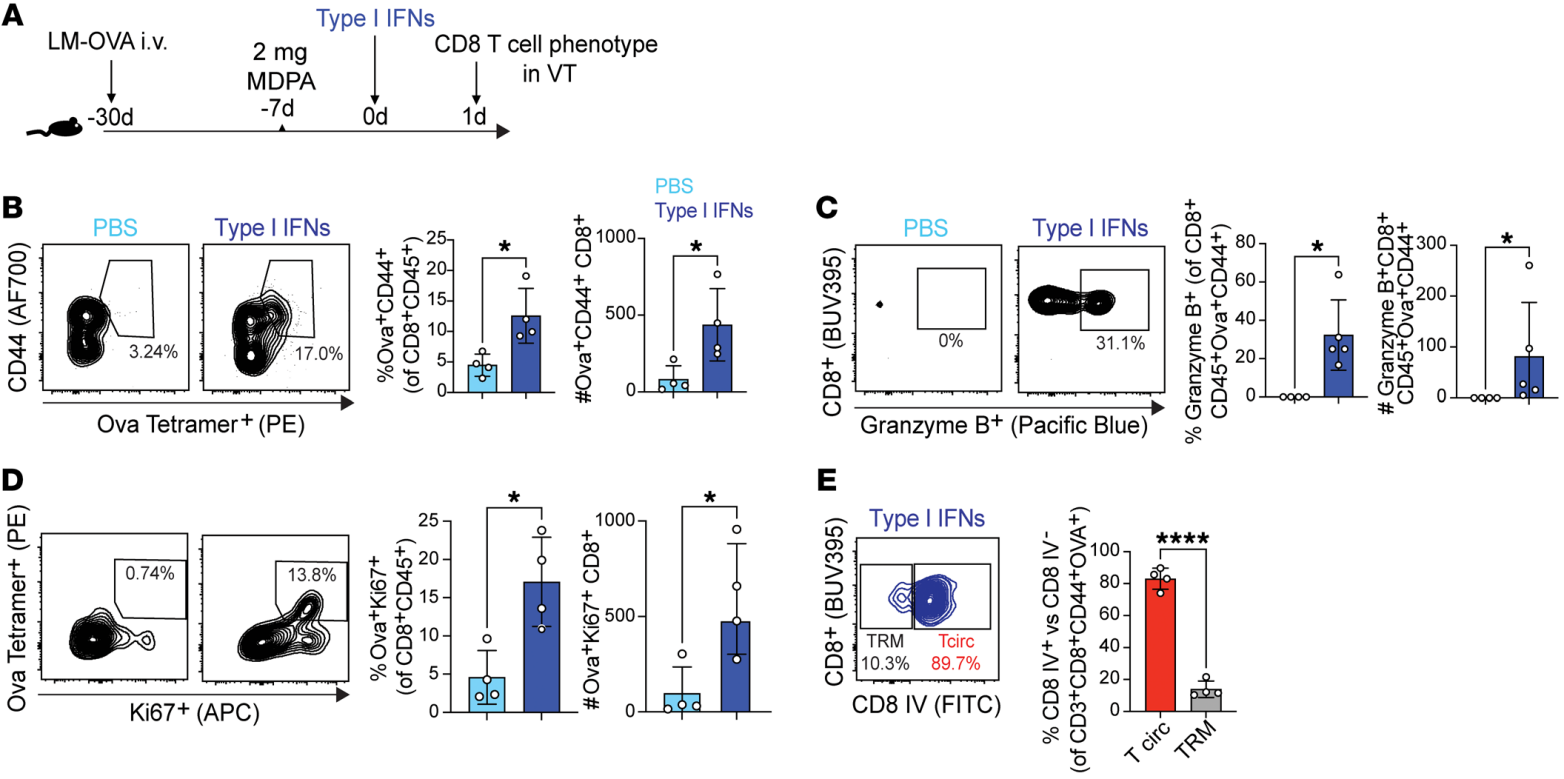
Vaginal memory CD8^+^ T cells increase in numbers and function in vivo upon type I IFN treatment. (**A**) Experimental schematic to assess CD8^+^ T cell phenotype after intravaginal treatment with type I IFNs in LM-OVA–immunized mice. (**B**) Representative flow plots and graphs to assess OVA tetramer–positive subset in the VT in LM-OVA–immunized mice that received either PBS alone or IFN-α/β. (**C**) Representative flow plots and graphs of granzyme B–expressing CD62L^–^ CD8^+^ T cells within the OVA tetramer–positive population. (**D**) Representative flow plots and graphs showing the OVA tetramer– and Ki67-positive population plotted on total CD8 gate. (**E**) Mice received intravenous injection with anti-CD8 antibody (CD8 IV) 5 minutes before euthanasia. OVA^+^CD44^+^ population was categorized as circulating T cells (CD8 IV^+^) and TRM (CD8 IV^–^) by intravascular staining. Each dot represents an individual mouse, and data are representative of at least 2 experiments with 4–5 mice per group. Error bars represent mean ± SD. Statistical significance was determined by Mann-Whitney test. *P < 0.05, ****P < 0.0001.

Next, we asked whether local administration of cytokines alone can result in the recruitment and activation of bystander T cells in the FGT in the absence of TRM interaction with cognate antigen. Previous studies indicate that type I IFN stimulation in mice mimics viral challenge, leading to a transient expansion of memory CD44-high cells (8, 38). Type I IFNs have also been shown to directly act on CD8^+^ T cells to provide survival signals (39) and can mediate an indirect effect on memory CD44-high CD8^+^ T cells via IL-15 production (40). Hence, we sought to determine whether vaginal treatment with type I IFN would be sufficient to elicit accumulation and activation of bystander T cells in the vaginal tract, which would demonstrate a truly TCR-independent, innate immune–driven activation of mucosal memory CD8^+^ T cells. LM-OVA memory mice received type I IFNs (α/β) or PBS via intravaginal delivery (Figure 5A). We assessed bystander T cells by gating OVA-specific cells expressing CD44 one day after local cytokine delivery (Figure 5B). We found that local delivery of type I IFN was sufficient to drive an increase in the number of vaginal OVA-specific T cells (Figure 5B). To examine the function of these memory CD8^+^ T cells and assess bystander activity, we measured granzyme B expression by OVA^+^CD44^+^ CD8^+^ T cells and found an expansion of granzyme B^+^ effector CD8^+^ T cells (Figure 5C). Additionally, there was a significant increase in the frequency and number of Ki67^+^ OVA-specific CD8^+^ T cells (Figure 5D), demonstrating that local type I IFN treatment is sufficient to induce proliferation of mucosal memory CD8^+^ T cells. Finally, to distinguish whether the OVA-specific population in the vaginal tract was present in the tissue parenchyma or the vasculature, we administered intravascular CD8 staining antibody (41). More than 80% of OVA-specific CD8^+^ T cells in the vaginal tract appeared to be within the vasculature based on intravascular label staining, confirming that cytokine treatment leads to trafficking of CD8^+^ T cells from the circulation and is not solely due to in situ proliferation of TRMs in the tissue (Figure 5E). Taken together, these findings demonstrate that murine memory CD8^+^ T cells can become bystander-activated in the mucosal tissues in the absence of cognate antigen upon local administration of type I IFN.

### Cytokine exposure is sufficient to induce a bystander-activated phenotype in circulating and tissue memory CD8^+^ T cells.

**Figure 6:**
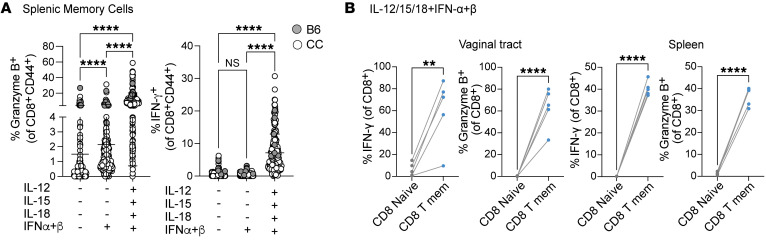
Memory CD8^+^ T cells acquire a bystander phenotype upon cytokine exposure. (**A**) Splenocytes from C57BL/6 (gray dots) and Collaborative Cross (CC-RIX) mice (white dots) were cultured in vitro for 24 hours with IFN-α/β (1,000 U) alone or with IL-12/15/18 (100 ng/mL). Granzyme B and IFN-γ expression was assessed within activated memory CD8^+^ T cells (CD44^+^) gated by flow cytometry. (**B**) Vaginal cells and splenic cells from LM-OVA memory mice were cultured in vitro for 24 hours with IFN-α/β (1,000 U) and IL-12/15/18 (100 ng/mL). IFN-γ and granzyme B expression within naive (CD44^–^) and memory CD8^+^ T (CD44^+^) cell populations was measured. Each dot represents an individual mouse, and data are representative of at least 2 experiments with 4–10 mice per mouse strain. Error bars represent mean ± SD. Statistical significance was determined by 1-way ANOVA with Tukey’s multiple comparisons (**A**) or unpaired *t* test (**B**). **P < 0.01, ****P < 0.0001.

Since HSV infection induces cytokines and generates a localized inflammatory response, we next wanted to understand the underlying mechanism of bystander activation and whether cytokines induced by HSV-2 infection could lead to activation of recruited memory CD8^+^ T cells. Thus, we performed ex vivo stimulation assays wherein we incubated naive splenocytes from C57BL/6J (B6) mice with cytokines to examine their effect on CD8^+^ T cell function. We first tested varying combinations of cytokines, such as type I IFN and IL-12, known to be induced upon mucosal HSV-2 infection and found in vaginal secretions (42), and IL-15 and IL-18, which are known to trigger bystander activation and to be present in the vagina after HSV-2 infection (43–45) (Supplemental Figure 6). We found that a combination of type I IFN and IL-12, -15, and -18 triggered a robust bystander activation phenotype in CD8^+^ T cells from B6 mice (gray dots), as noted by IFN-γ and granzyme B production (Figure 6A). To recapitulate the genetic diversity in humans and to rule out the possibility of strain-specific differences, we used splenocytes from genetically diverse mice from the Collaborative Cross (CC) (46, 47) and compared the granzyme B and IFN-γ response with that in B6 mice after cytokine stimulation (Figure 6A). While we found a considerable range in the responses based on genetic background, stimulation with IL-12/15/18 plus type I IFNs led to a significant increase in both IFN-γ and granzyme B production by memory CD8^+^ T cells (Figure 6A). Further, we found that stimulation with type I IFN alone led to a significant increase in the frequency of CD8^+^ T cells expressing granzyme B while IFN-γ levels remained unaltered (Figure 6A).

Next, we wanted to determine whether cytokines can similarly activate memory CD8^+^ T cells from the vaginal mucosa. Bulk vaginal and splenic single-cell suspensions from LM-OVA memory B6 mice were cultured overnight with IL-12/15/18 and type I IFN, and GolgiStop (BD Biosciences) was added the next day for the final 4 hours to measure intracellular IFN-γ and granzyme B. To generate a memory population within the vaginal tract, we immunized mice with LM-OVA and compared intracellular cytokine responses in LM-OVA–immunized mice within naive (CD44-low) and memory (CD44-high) populations. As with splenic memory CD8^+^ T cells from B6 mice, the addition of cytokines led to a significant increase in IFN-γ and granzyme B production in the vaginal tract (Figure 6B), suggesting that while naive memory CD8^+^ T cells are unresponsive to the cytokines, memory CD8^+^ T cells from mucosal tissues can be bystander-activated without the requirement of TCR stimulation in the context of cytokine treatment. Finally, given that virtual memory (VM) CD8^+^ T cells are also sensitive to cytokine treatment, we assessed whether cytokine stimulation led to a significant increase in IFN-γ and granzyme B production within the CD8^+^ VM population. The VM subset from bulk splenic single-cell suspensions from B6 mice was gated as CD49d^–^CD122^+^CD44^+^ (Supplemental Figure 7A). Overall, we found that the CD8^+^ T cell compartment within naive unimmunized B6 mice consisted of 10%–20% VM cells (Supplemental Figure 7A). As in the general CD44^+^ CD8^+^ memory subset, treatment with IL-12/15/18 and type I IFN resulted in significantly more intracellular IFN-γ and granzyme B expression within the VM population (Supplemental Figure 7, B and C).

### Human mucosal tissue memory CD8^+^ T cells acquire a bystander phenotype upon cytokine treatment.

**Figure 7:**
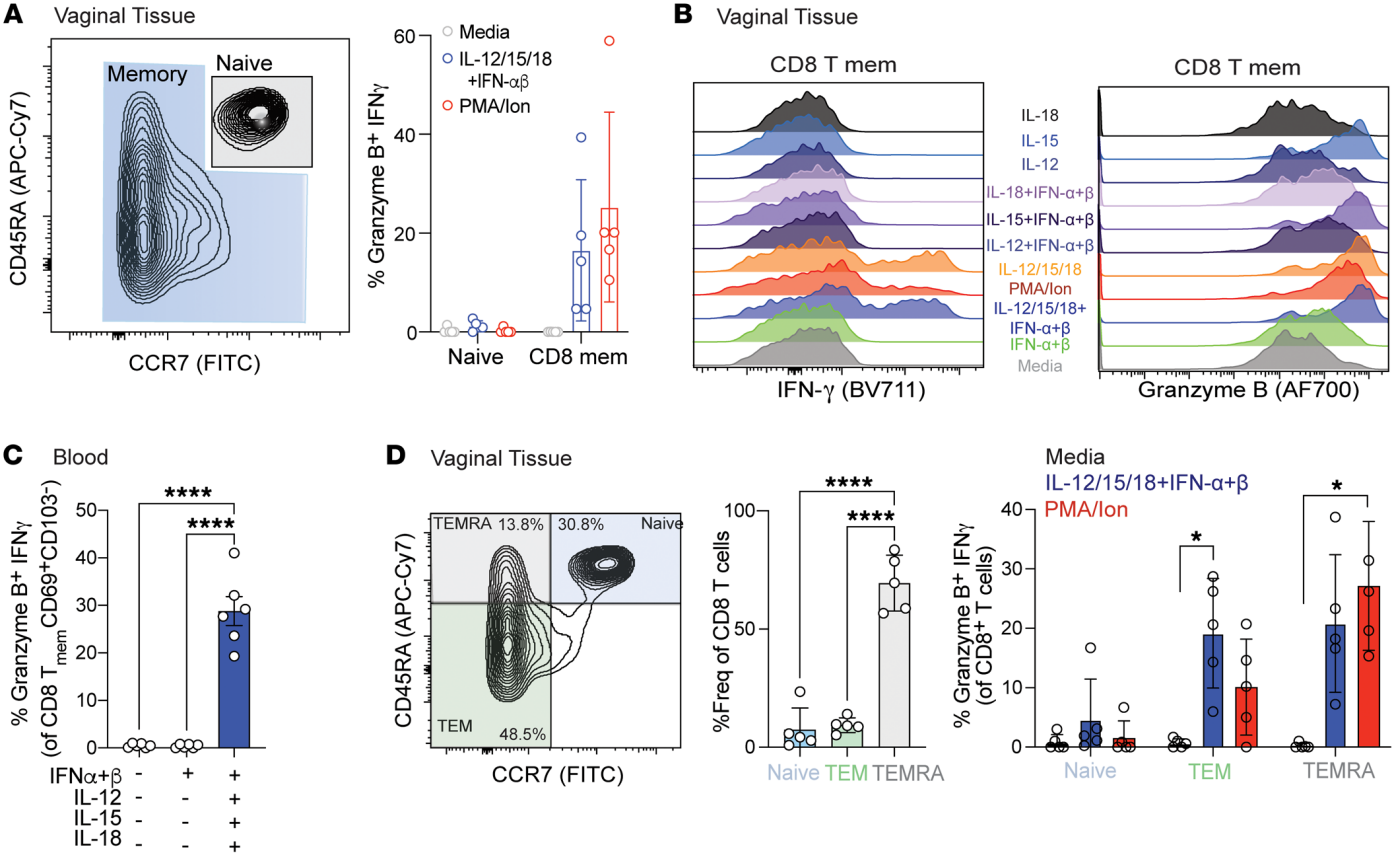
Human memory CD8^+^ T cells in the vaginal tissue acquire bystander phenotype upon cytokine treatment. Cells from vaginal tissues obtained from prolapse repair surgeries were cultured in vitro for 24 hours with varying combinations of IFN-α/β (1,000 U) and IL-12/15/18 (100 ng/mL). (**A**) Left: Representative flow plot shows the distribution of CD8^+^ T cells based on CD45RA and CCR7 markers highlighting memory (blue) and naive (gray) compartments. Right: Graph plot shows coexpression of granzyme B and IFN-γ within each compartment after treatment with medium or cytokines. Each dot represents an individual condition, and the color code for each condition is the same as in **B**. (**B**) Staggered histogram separated per different cytokine treatments showing IFN-γ and granzyme B expression within the memory CD8^+^ T cell compartment. (**C**) Human PBMCs were cultured for 24 hours with IFN-α/β (1,000 U) alone or with IL-12/15/18 (100 ng/mL). Graph plots show memory CD8^+^ T cells expressing granzyme B and IFN-γ with varying cytokine combinations. (**D**) Flow plot shows the distribution of CD8^+^ T cells as naive (CCR7^+^CD45RA^+^), TEM (CCR7^–^CD45RA^–^), and TEMRA (CCR7^–^CD45RA^+^). Graph plot represents each CD8^+^ T cell subset within an individual donor followed by expression of granzyme B and IFN-γ within each subset. Each dot represents an individual donor, and data are pooled from 5 separate donors. Error bars represent mean ± SD. Statistical significance was determined by 1-way ANOVA with Tukey’s multiple comparisons. *P < 0.05, ****P < 0.0001.

Finally, to address whether human memory CD8^+^ T cells are similarly activated as compared with mouse CD8^+^ T cells after cytokine stimulation, we obtained vaginal tissues from healthy women undergoing vaginal repair surgeries. Single-cell suspensions were first prepared and cultured with several cytokine combinations ex vivo, followed by measurement of IFN-γ and granzyme B production. The CD8^+^ T cell compartment was distinguished based on CD45RA and CCR7 expression into naive and memory subsets (Figure 7A and Supplemental Figure 8). As expected, the naive CD8^+^ T cell subset remained unaltered despite cytokine exposure. In contrast, the memory CD8^+^ T cells from human vaginal tissues expressed granzyme B and IFN-γ upon stimulation with cytokines or PMA/ionomycin (Figure 7A). We found that a combination of IL-12, IL-15, and IL-18 led to increased IFN-γ and granzyme B levels, whereas IL-15 alone or IL-15 with type I IFN induced an elevated granzyme B response (Figure 7B). Likewise, circulating CD8^+^ T cells in human PBMCs were similarly activated after cytokine exposure (Figure 7C). We further divided the tissue memory compartment into effector memory T cells (TEM), terminally differentiated effector memory cells (TEMRA), and naive cells and compared their activation profile after cytokine exposure (Figure 7D). While TCR stimulation via PMA/ionomycin resulted in higher granzyme B and IFN-γ in the TEMRA subset, treatment with cytokines caused more activation in TEM cells. Finally, we assessed two CD8^+^ T cell populations: tissue resident (CD69 and CD103 coexpression) and activated (CD69 expression alone). Both TRMs and the CD69^+^ population produced significantly more granzyme B and IFN-γ after stimulation with IL-12/15/18 (Supplemental Figure 9). Contrary to murine cells, intracellular granzyme B levels remained unaltered after exposure with type I IFNs alone. In summary, our data from both mouse and human tissues suggest that mucosal memory CD8^+^ T cells are sensitive to cytokine treatment, and the provision of cytokines alone is sufficient to trigger memory CD8^+^ bystander activation.

## Discussion

Due to their history of immune experience, humans have a significant pool of memory CD8^+^ T cells at barrier surfaces, which remain at these pathogen portals of entry. Vaccine strategies aimed at eliciting CD8^+^ TRMs have gained considerable interest due to their role in pathogen clearance since they are retained in barrier tissues and are thus among the first immune cells to encounter pathogens (30, 48). A recent mathematical modeling study on human HSV-2 reactivation and shedding suggests that a low density of CD8^+^ TRMs in the genital mucosa could recruit antigen-independent BA-CTLs and thereby contribute to the rapid clearance of the virus (29). Therefore, both antigen-specific and -nonspecific CD8^+^ T cells are likely to be essential elements of an effective immune response. Here, we provide experimental evidence that supports this in silico model; we used a mouse model of genital HSV-2 to regulate and track the memory CD8^+^ T cell compartment to dissect the activation cues and functionality of BA-CTLs in the mucosal tissue. We report that memory CD8^+^ T cells generated by immunization with an irrelevant antigen partially protected mice from vaginal HSV-2 infection without the requirement of cognate antigen interaction (Figure 1). We found that granzyme B and NKG2D expression was restricted to the tissue, suggesting that local environmental cues in the mucosal tissue can activate memory CD8^+^ T cells in a TCR-independent manner, and hinting at a mechanism by which BA-CTLs could mediate target cell killing (Figure 2). It has previously been shown that TRMs in the FGT and skin mediate broad antiviral effects via cytokine-dependent mechanisms (13, 20). However, cognate peptide interaction prior to infection with an irrelevant pathogen was critical to lowering the viral burden (1, 2). Based on our findings, we conclude that antigen-nonspecific memory CD8^+^ T cells do not rely on cognate peptide interactions to facilitate improved clinical outcomes upon vaginal HSV-2 infection (Figure 1). This finding is in line with the report by Ge et al. (17) that bystander activation of lung TRMs attenuated the severity of pulmonary infection. However, we demonstrate that, contrary to lung infection, even though bystander T activation occurs in the inflamed vaginal tract (Figure 2), bystander T cells are not necessarily tissue-resident in origin, but rather many of these cells appear to migrate to the site of localized infection to become bystander-activated (Figure 3). Thus, we propose that memory CD8^+^ T cells from secondary lymphoid organs or the circulating pool mobilize to the tissue site and become bystander activated, without TCR engagement (Figure 2F), to participate in early, innate-like viral clearance.

The study by Raué determined the importance of cytokine-stimulated antigen-specific CD8^+^ T cells from LCMV-immune mice leading to an increase in in vivo proliferation and reduction in viremia (5). Here, we made a surprising observation that even very few cytokine-activated memory CD8^+^ T cells or even preexisting memory CD8^+^ T cells from a previous immunization with irrelevant antigen specificity can initiate an alarm signal and are capable of reducing the viral burden upon infection (Figure 4 and Supplemental Figures 3 and 4). Since the phenomenon of bystander activation is inflammation driven, we looked at the underlying mechanism of whether memory CD8^+^ T cells can be activated in an antigen-free setting. Type I IFNs have also been shown to act on memory CD8^+^ T cells directly or indirectly via IL-15 signaling to provide survival signals (39, 40). We found that administration of type I IFNs led to an antigen-free inflammation state resulting in bystander activation and accumulation of memory CD8^+^ T cells in the FGT and upregulated granzyme B expression. This indicates that type I IFN, known to be potently induced locally following many viral infections, including HSV-2, is sufficient to trigger bystander activation of memory CD8^+^ T cells within the mucosa, without the need for TRM recognition of cognate peptide. In contrast, the bystanders in the vaginal tract could be trafficking to the vaginal mucosa after encountering inflammation or cytokines as a by-product of infection (Figure 5). Based on our in vivo and in vitro results, we found that treatment with type I IFNs leads to increased abundance of granzyme B–producing innate-like memory cells in the vagina. However, type I IFNs alone are unable to activate human memory CD8^+^ T cells (Figure 7). It is possible that type I IFNs act directly on memory CD8^+^ T cells via IFNAR or may trigger an indirect cascading effect on other cells such as DCs, which may lead to production of IL-15 or other cytokines, which could in turn act on the common γ chain receptor on memory CD8^+^ T cells (40). Further investigation is required to determine the cellular mechanism of type I IFN and whether local treatment with type I IFN causes direct or indirect bystander activation of memory CD8^+^ T cells in the FGT, and to distinguish the involvement of cytokine receptors expressed on memory CD8^+^ T cells that are important in bystander activation. A vaccination strategy called “prime and pull” shows that subcutaneous administration of HSV-tk (an attenuated strain of HSV, described as the “prime”) and subsequent topical administration of the chemokines CXCL9 and CXCL10, described as “pull,” to recruit HSV-specific T cells at the site of infection protects against genital HSV-2 infection (49). This strategy relies on CD8^+^ T cells to mediate protection, as the deletion of CD8^+^ T cells in these mice prevented protection (26). Hence, we propose a model wherein localized inflammation resulting from type I IFNs could serve as a “pull” mechanism to recruit peripheral polyclonal memory CD8^+^ T cells to the tissue to thus participate in very early and innate-like immune protection in the mucosa in a TCR-independent fashion. Additionally, chemokines and their receptors may be involved in cytokine-induced tissue homing of bystander-activated T cells, and it remains possible that memory CD8^+^ T cells must be restimulated in the lymph node before the accumulation of bystander-activated CD8^+^ T cells in the infected mucosa. Previous studies show that bystander T cells rely on CXCR3 to migrate rapidly to the site of localized inflammation (20). CXCR3 ligands such as CXCL9 and CXCL10 also cause bystander T cell migration in the case of Lyme disease, causing inflammation in joints and arthritis (16). Additionally, CCR5 has been reported to be upregulated by IL-15, causing migration of bystander CD8^+^ T cells to the infection site to mediate enhanced liver injury in acute hepatitis A (50).

Since bystander activation is primarily mediated by inflammatory cytokines (4, 11, 51), we looked at combinations of cytokines such as type I IFNs, IL-12, IL-15, and/or IL-18, as these are known to activate memory CD8^+^ T cells to become bystander-activated and are associated with improved HSV control. In addition to mediating systemic proliferation and expansion of polyclonal memory CD8^+^ T cells, type I IFNs signal through the type I IFN receptor (IFNAR-1) and cause antigen-independent expression of granzyme B (11). IL-12 and -18 induce IFN-γ production, possibly contributing to early infection control, whereas IL-15 equips memory CD8^+^ T cells to produce cytotoxic molecules like granzyme B and perforin (10, 11, 14). We found that stimulation with IL-12, IL-15, IL-18, and type I IFNs resulted in IFN-γ and granzyme B secretion by memory CD8^+^ T cells from human and mouse vaginal tissues (Figures 6 and 7). We found that treatment with IL-15 alone elicited granzyme B expression (Figure 7). Our study supports the work by Kohlmeier et al. (11), which demonstrated that antigen-nonspecific memory CD8^+^ T cells express granzyme B and possess enhanced cytolytic potential when recruited to the lung during respiratory viral infection. However, their study did not clarify the role of the recently recruited cells in reducing pathogen burden. Furthermore, this previous work showed a correlation in granzyme B expression in the airways and distal sites. Uniquely, our study demonstrates that the bystander potential of memory CD8^+^ T cells is restricted to the location of infection (Figure 2), as seen in a study by Maurice et al. (20). Our finding is consistent with a potential immunoregulatory mechanism that restricts TCR-independent activation of memory T cells that are not located in active tissue sites of inflammation, such as the spleen and draining lymph nodes during a localized mucosal infection. Further, we demonstrate a reduction in early local viral burden and clinical symptoms mediated by bystander-activated CD8^+^ T cells (Figure 4), thereby demonstrating an innate-like protective role of BA-CTLs in mucosal tissue protection following virus infection. How these BA-CTLs mediate partial protection remains unresolved, though several possibilities exist. In addition to granzyme B, we also found enhanced NKG2D expression within vaginal antigen-irrelevant CD8^+^ memory T cells. CD8^+^ T cells can achieve targeted delivery of cytotoxic molecules via NKG2D-mediated immune synapse formation, which could be one of the killing mechanisms by which these cells could kill infected cells (4). The BA-CTLs could also exert heightened cytotoxicity and potentially recruit more innate cells by relying on IFN-γ–dependent mechanisms to achieve target cell killing in an autocrine or paracrine manner (4, 52).

The ability of polyclonal memory CD8^+^ T cells to become activated in barrier tissue sites upon type I IFN signaling could present the host with a dangerous situation if left uncontrolled. A previous study indicated that exposure to poly I:C led to short-term proliferation of memory CD8^+^ T cells (up to 3 days) (7), suggesting a potentially transient nature of bystander activation. The short-lived activation status of BA-CTLs during HSV-2 infection could benefit the host by providing a short-lived innate immune–type protection that wanes quickly before mediating immunopathology. Hence, the duration of the activation status and the regulatory mechanisms that keep BA-CTLs in check warrant further investigation.

Overall, our study reveals that memory CD8^+^ T cells with irrelevant antigen specificity can act in a rapid, innate-like manner upon recruitment to inflamed tissue sites to provide early mucosal immune protection during virus infection. We explored bystander T cells’ relevance in mucosal infection to gain mechanistic insights into their activation and functionality. To our knowledge, this is the first study to show the cytokine sensitivity of FGT memory CD8^+^ T cells and demonstrate the significance of TCR-independent, innate-like memory CD8^+^ T cells in limiting early viral burden in the context of mucosal infection within the FGT. The findings increase our understanding of how antigen-irrelevant memory CD8^+^ T cells may enhance the immunogenic response and shed light on the settings in which they can be leveraged to be beneficial for design of vaccines and prophylactics.

## Methods

### Mice.

Six- to eight-week-old female C57BL6/J or Nur77-GFP reporter mice were purchased from The Jackson Laboratory (strains 000664 and 016617) and maintained in specific pathogen–free conditions at the Fred Hutchinson Cancer Research Center. Male and female Collaborative Cross (CC) mice were purchased from the Systems Genetics Core Facility at the University of North Carolina at Chapel Hill. These mice were euthanized in the laboratory of M.T. Ferris at 6–8 weeks of age, and spleens were then shipped to the J.M. Lund laboratory. The CC strains were used as described in Supplemental Table 1.

### L. monocytogenes immunization.

To generate LM-OVA or LM-OVA-gB memory mice, mice received intravenous immunization with *Listeria monocytogenes* strains that were generated to recombinantly express and secrete OVA with or without the HSV-2 glycoprotein B–derived (gB-derived) peptide SSIEFARL according to previously described methods. The pPL2 vector was used to achieve integration into the bacterial genome (25, 53, 54). The mice received 4,000 CFU of LM-OVA or LM-OVA-gB and were rested for 30 days after immunization before they were infected with wild-type HSV-2.

### Vesicular stomatitis virus immunization.

Mice received 1 × 10^6^ PFU of OVA-expressing vesicular stomatitis virus (VSV-OVA) (55) in sterile PBS and were rested for at least 60 days after immunization before they were infected with wild-type HSV-2.

### HSV-2 infection, clinical scoring, and PCR.

Mice were subcutaneously injected with 2 mg of medroxyprogesterone acetate (MDPA) injectable suspension (Depo-Provera) dissolved in sterile PBS 5–7 days before vaginal infection. For HSV-2 infection, mice were infected intravaginally with 10^4^ PFU of wild-type (186syn+) HSV-2 (25, 56). Disease severity was assessed using a pathology scoring system as follows: 0, no sign; 1, slight genital erythema; 2, moderate genital erythema and edema; 3, significant genital inflammation with visible lesion; 4, hind leg paralysis or other severe condition requiring euthanasia; and 5, moribund or dead. Mice were euthanized if they were moribund or showed signs of severe disease, including hind leg weakness, hind leg paralysis, 20% decline in weight, or hunched posture. Vaginal swabs were collected in 200–1,000 μL of titration buffer (0.05 g MgCl_2_∙6H_2_O, 0.066 g 2H_2_O, 5 g glucose, 5 mL FBS dissolved in 500 mL PBS). The vaginal canal was swabbed thoroughly with a Puritan calcium alginate swab twice, followed by washing of the vaginal canal with 50 μL of titration buffer. Viral titer was determined by PCR as previously described (57).

### Mouse tissue collection and processing.

The vaginal tract and cervix were harvested and minced thoroughly in prewarmed digestion medium, which was freshly prepared and consisted of DMEM, collagenase D (2 mg/mL; Sigma-Aldrich), 500 μL DNase (15 μg/mL), and 10% FBS. The minced tissue was incubated for 30 minutes at 37°C on a shaker. After incubation, the minced tissue was spun at 300 *g* for 5 minutes. To prepare single-cell suspensions, 5 mL of HBSS/EDTA solution (2% FBS in HBSS without calcium and magnesium and 5 mM EDTA) was added to the pellet, and the mixture was mashed through a 70 μm strainer. Draining lymph nodes (iliac and inguinal) were collected in RP10 medium (RPMI 1640 with 10% FBS, 2 mM l-glutamine, 100 U/mL penicillin-streptomycin, 5 mM sodium pyruvate) and mashed through a 100 μm filter. Spleens were collected in RP10 medium and mashed through a 100 μm filter, and the cells were centrifuged at 300 *g* for 5 minutes. The pellet was resuspended in 5 mL ACK lysis buffer for 2 minutes and centrifuged at 300 *g* for 5 minutes. The ACK-treated cells were suspended in RP10 medium before proceeding with flow staining.

### Human tissue collection and processing.

Vaginal tissues were obtained from vaginal repair surgeries at the University of Washington Medical Center. RP15 (RPMI 1640 with 15% FBS, 2 mM l-glutamine, 100 U/mL penicillin-streptomycin) and digestion medium were freshly prepared. The digestion medium consisted of collagenase type II (C6885, Sigma-Aldrich) dissolved at 700 collagen units/mL and 1 U/mL DNase I (Sigma-Aldrich) in a 1:1 mixture of PBS and R15 as described in ref. 58 and was warmed for 30–90 minutes in a 37°C bead bath before use. Vaginal tissues were washed with PBS, and the underlying tissue was trimmed off, leaving 2 mm of epithelium and the outermost stroma. The tissue was minced and pipetted into a 50 mL Falcon tube with 20 mL digestion medium and shaken for 30 minutes at 37°C in a shaker incubator. The collagenase-treated sample was then aspirated and expelled from a 30 mL syringe through a 16-gauge blunt needle 10 times and then strained through a 70 μm strainer. The cell suspension was stored on ice. The remaining tissue chunks were digested again in fresh digestion medium for 30 minutes at 37°C in a shaker incubator and then passed through the needle and strained. The cell suspensions were mixed and pelleted in a centrifuge at 300 *g* for 5 minutes at 22°C and resuspended in fresh RP10 medium. Cells (0.5 × 10^6^ to 1 × 10^6^) were seeded in a 96-well plate in RP10 supplemented with a combination of cytokines or PMA (50 ng/mL)/ionomycin (500 ng/mL) as follows: recombinant human IL-12, 200 ng/mL (PeproTech, catalog 200-12H, lot 0918711); recombinant human IL-15, 200 ng/mL (PeproTech, catalog 200-15, lot 022124); recombinant human IL-18, 100 ng/mL (MBL International, catalog B003-5, lot 091); human IFN-α, 500 U (PBL Assay Science, catalog 11200-1, lot 7155); human IFN-β, 500 U (PBL Assay Science, catalog 11415-1, lot 7349). GolgiPlug (BD Biosciences) was added at a dilution of 1:1,000 four hours before cell harvesting.

### Cell staining for flow cytometry.

Cells were incubated in Live/Dead Blue fixable amine-reactive viability dye (Thermo Fisher Scientific) for 25 minutes at room temperature. The flow panels that were used to characterize the immune cells are described in Supplemental Tables 2 and 3 (mouse) and Supplemental Table 4 (human). Cytosolic and nuclear proteins were detected using Foxp3/Transcription Factor Fixation/Permeabilization reagents (eBioscience). Samples were acquired on a FACSymphony instrument (BD Biosciences). For in vivo T cell labeling, CD8b monoclonal antibody (FITC; Thermo Fisher Scientific, clone: H35-17.2, catalog 11-0083-82) was administered via an intravascular route by retro-orbital injection 5 minutes before the mice were euthanized.

### Intravaginal cytokine treatment.

LM-OVA memory mice received IFN-α (12100-1, PBL Assay Science) and IFN-β (12400-1, PBL Assay Science) in a 10 μL volume via the intravaginal route. Each mouse received a total of 2,000 U of each cytokine. The vaginal canal was swabbed to remove mucus before the cytokine administration.

### Treatment with FTY720 and CD8 depleting antibody.

LM-OVA memory mice received injections of 1 mg/kg FTY720 (Sigma-Aldrich) dissolved in 2% cyclodextrin (diluted in sterile PBS) via an intraperitoneal route on days –1, 0, +1, and 2 relative to HSV-2 infection. Control mice received diluent (2% cyclodextrin in sterile PBS).

CD8^+^ T cells were depleted at days –3, –1, +1, and +3 relative to HSV-2 infection by intraperitoneal injection of 200 μg of anti-CD8 (BE0061, Bio X Cell) or isotype control antibody (BE0090, Bio X Cell) in 200 μL volume (diluted in sterile PBS).

### In vitro culture.

Single-cell suspensions were obtained by mashing of spleens with a 3 mL syringe plunger, followed by ACK treatment. Cells (1 × 10^6^) were plated in a 96-well plate and spun down at 300 *g* for 5 minutes. The supernatant was carefully removed, and RP10 medium containing a combination of cytokines was added: recombinant mouse IL-12, carrier-free (577004, BioLegend) at 100 ng/mL; recombinant mouse IL-15, carrier-free (566302, BioLegend) at 100 ng/mL; recombinant mouse IL-18, carrier-free (767004, BioLegend) at 0.25 ng/mL; mouse IFN-α A and IFN-β (12100-1, 12400-1, PBL Assay Science) at 1,000 U/mL.

### Adoptive transfers.

CD8^+^ T cells were purified from spleens and lymph nodes obtained from naive and LM-OVA–immunized CD45.1 donor mice using a mouse CD8^+^ T cell isolation kit (19853, Stemcell Technologies). CD8^+^ T cells from naive mice were stimulated with medium or IL-12+15+18 (10 ng/mL) plus type I IFNs (500 U each) for 5 hours. The supernatant was collected for IFN-γ ELISA (KMC4021C, Thermo Fisher Scientific). Cells were washed with PBS and transferred in CD45.2 recipient mice via an intravenous route. Each mouse received 1 × 10^6^ cells. For the adoptive transfer of CD44^+^ memory CD8^+^ T cells and CD44^–^ naive CD8^+^ T cells, CD8^+^ T cells were purified from spleens and lymph nodes obtained from LM-OVA–immunized CD45.1 donor mice using a mouse CD8^+^ T cell isolation kit (Stemcell Technologies). To purify CD44-expressing population, purified CD8^+^ T cells were incubated with anti-CD44–PE beads (130-118-566, Miltenyi Biotec) for 15 minutes, followed by incubation with anti-PE microbeads (130-048-801, Miltenyi Biotec) for 15 minutes. The cells were passed through the LS column (Miltenyi Biotec) per the manufacturer’s instruction. The eluted negative fraction was CD44^+^ cells, whereas the positive fraction in the column was CD44^–^ cells eluted using a plunger. Cells were washed with PBS and transferred to CD45.2 recipient mice via an intravenous route. Each mouse received 2 × 10^6^ cells.

### Statistics.

All statistics were performed using Prism software (GraphPad Software). Unpaired 2-tailed *t* tests were used to compare the means between 2 groups. For comparison of more than 2 groups, 1-way ANOVAs with Tukey’s multiple-comparison test were used. Two-way ANOVAs were used for clinical scoring data analysis. Log-rank tests were used to analyze survival curves. The error bars represent standard deviation, and significance was defined as *P* less than 0.05.

### Study approval.

Mouse experiments were approved by the Fred Hutchinson Cancer Research Center Institutional Animal Care and Use Committee (IACUC). Experiments involving CC mice were approved by the University of North Carolina at Chapel Hill IACUC. Human vaginal tissues, which would otherwise be discarded, were obtained from vaginal repair surgeries at the University of Washington Medical Center. These tissues were obtained under a waiver of consent approved by the University of Washington IRB (no. 1167). The study was conducted according to the principles of the Declaration of Helsinki.

## Author contributions

TA performed all the experiments. VD, ICT, and JLS assisted in tissue processing and intravenous injections and served as blinded scorers for HSV clinical scoring. JBG and JLS processed spleens from the Collaborative Cross mice and performed experiments in Nur77-GFP reporter mice. TAB, PH, JF, and GDS collected spleens from CC mice. SMH, AK, and MF collected the human clinical samples. SMH assisted in tissue processing. MLH performed real time PCR analysis. MP provided the *Listeria* constructs. KRJ, MTF, FH, JTS, MP, and JML supervised and mentored staff and contributed to study design. TA and JML conceptualized the study and wrote the first draft of the manuscript. All the authors contributed to editing and approved the final draft.

## Supplementary Material

Supplemental data

## Version 1 03/23/2023

In-Press Preview

## Version 2 05/15/2023

Electronic publication
