# Supplementary material for: Memory T cells possess an innate-like function in local protection from mucosal infection
Source: J Clin Invest. 2023 May 15;133(10):e162800. doi: 10.1172/JCI162800 (PMC10178838; doi:10.1172/JCI162800)
Supplement: Supplemental data [file jci-133-162800-s070.pdf]

Supplementary Material

Supplemental Figure Legends

Supplemental Figure 1. Antigen non-specific CD8 T cells are present in the vaginal tract upon HSV-2 infection in mice immunized with LM-OVA-gB. (A) Experimental schematic to generate HSV gB-specific and OVA-specific memory compartment by immunizing mice with LM-OVA-gB followed by intra-vaginal challenge with wild-type HSV-2. Mice were euthanized on days 1-3 after-WT HSV-2 challenge to assess gB-and OVA-specific CD8 T cells in the vaginal tract (VT). (B) Representative gating scheme of vaginal tract (VT) to assess CD8 T cells on day 2 post-HSV-2 challenge. (C) Percent frequency and counts of OVA-tetramer and gB-tetramer cells on days 1-3 post-HSV-2 challenge in LM-OVA-gB immunized mice. Each dot represents an individual mouse and data is representative of two experiments with 4-5 mice per group. Error bars represent mean ± SD.

Supplementary Figure 2. LM-OVA immunized mice show exacerbated disease and higher HSV viral titers after CD8 Depletion. (A) Experimental schematic to compare protective efficacy of LM-OVA (HSV- non-specific) immunization after CD8 depletion. CD8 T cells were depleted by i.p route with 200 μg anti-CD8 antibody or isotype antibody on indicated days relative to the day of WT HSV-2 infection. (B) Confirmation of depletion of vaginal CD8 T cells (Day 2 p.i). Mice were monitored for clinical score (C) and survival (D) after lethal HSV-2 challenge, and viral titers were determined by RT-PCR on days 1, 2, 3, and 6 p.i. by collecting vaginal washes (E). Each dot represents an individual mouse and data is pooled from two to three experiments with 10-12 mice per group. Error bars represent mean ± SD. Statistical

significance was determined by Two-way-ANOVA, Tukey's multiple comparisons for (C), by Log-rank test for (D), and by unpaired t test in (B) and (E). * indicates $p<0.05$, ** indicates $p<0.01$, and *** indicates $p<0.001$.

Supplemental Figure 3. Adoptively transferred memory CD8 T cells from LM-OVA immunized mice delay the progression of lethal HSV-2 infection and lower the viral burden. (A) Experimental outline to compare the protective efficacy of mice receiving CD8 T cells from immunized and unimmunized mice. CD8 T cells were purified from splenocytes and draining lymph nodes and were derived from LM-OVA immunized or unimmunized donor mice. (B) Representative graph plots and bar graphs to assess the frequency of the CD44 expressing population in unimmunized and LM-OVA immunized mice within purified CD8 T cells before transfer. Mice were monitored for clinical score (C) and survival (D) after lethal HSV-2 challenge. (E) Vaginal washes were obtained after HSV-2 infection and viral titers were measured by RT-PCR on days 1, 2, 3 and 6 after HSV-2 infection. Data are pooled from at least two independent experiments with n=5-10 for the control and experimental group. Error bars represent mean ± SD. Statistical significance was determined by unpaired t test (B) and (E), Two-way-ANOVA, Tukey's multiple comparisons for (C) and by Log-rank test for (D). * indicates $p<0.05$, ** indicates $p<0.01$, and **** indicates $p<0.0001$.

Supplemental Figure 4. Mice with adoptively transferred memory CD44 high CD8 T cells from LM-OVA immunized mice display improved clinical scores after lethal HSV-2 infection. (A) Experimental outline to compare the protective efficacy of mice receiving CD44high or CD44low CD8 T cells from LM-OVA immunized mice. CD8 T cells were purified

from splenocytes and draining lymph nodes and were derived from LM-OVA immunized, 30 days after immunization. CD44 high or low cells were transferred via intravenous route and each mouse received 2 million cells at the time of HSV-2 challenge. Representative histogram plots to assess purity of CD44 high and CD44 low cells are shown. Mice were monitored for clinical score (B) and survival (C) after lethal HSV-2 challenge. (D) Vaginal washes were obtained after HSV-2 infection and viral titers were measured by RT-PCR on days 1, 2, 3 and 6 p.i. Error bars represent mean ± SD. Statistical significance was determined by Two-way-ANOVA, Tukey's multiple comparisons for (B), by Log-rank test for and for (C) unpaired t test for (D). *** indicates $p<0.001$.

Supplemental Figure 5. OVA-specific cells in the draining lymph nodes and spleens of LM-OVA memory mice. Percent frequency and counts of OVA-tetramer+ cells on days 1-3 post-HSV-2 challenge in LM-OVA immunized (blue) or unimmunized (gray) mice in spleen (A) and draining lymph nodes (inguinal and iliac) (B). Each dot represents an individual mouse and data are representative of two experiments with 4-5 mice per group. Error bars represent mean ± SD. Statistical significance was determined by One-way-ANOVA. * indicates $p<0.05$.

Supplemental Figure 6. HSV-2 infected mice exhibit elevated levels of IL-12, IL-15, and IL-18 levels in the vagina at 2 days post-infection. Mice were intravaginally infected with a lethal dose of wild-type HSV-2. Vaginal washes were obtained on day 2 after HSV-2 infection or from control naive mice. IL-12, IL-15, and IL-18 levels were measured by ELISA from the vaginal washes. Error bars represent mean ± SD. Statistical significance was determined by unpaired t-test. * indicates $p<0.05$ and ** indicates $p<0.01$.

Supplemental Figure 7. Virtual memory CD8 T (TVM) cells acquire a bystander phenotype upon cytokine exposure. (A) Representative gating strategy and percent frequency of virtual memory CD8 T cells among all CD8 T cells within splenocytes of unimmunized C57BL/6 mice. (B and C) Splenocytes from C57BL/6 were cultured in-vitro for 24 hours with media alone, IFN-alpha/beta (1000 U) or with IL-12/15 and 18 (100ng/mL). Granzyme B and IFN-γ expression was assessed within TVM population (CD8+CD49d-CD122+CD44+) gated by flow-cytometry. Each dot represents an individual mouse, and data is representative of at least two experiments with 4-8 mice. Error bars represent mean ± SD. Statistical significance was determined using One-way-ANOVA with Tukey's multiple comparisons. *** indicates $p<0.001$ and **** indicates $p<0.0001$.

Supplemental Figure 8. Gating strategy human vaginal tissue. Cells from vaginal tissues obtained from prolapse repair surgeries were cultured in-vitro for 24 hrs with media alone or varying combinations of IFN-alpha/beta (1000 U), IL-12/15, and 18 (100ng/mL). Representative gating strategy to assess memory CD8 T cell subsets based on CD45RA and CCR7 expression, tissue resident population based on CD69 and CD103 expression and granzyme B and IFN-γ expressing populations are shown.

Supplemental Figure 9. Both activated and tissue-resident memory CD8 T cells in the vaginal tissue acquire bystander phenotype upon cytokine treatment. Cells from vaginal tissues obtained from prolapse repair surgeries were cultured in-vitro for 24 hrs with varying combinations of IFN-alpha/beta (1000 U), IL-12/15, and 18 (100ng/mL). (A,B) Graph plots

show the frequency of CD69+ and CD69+CD103+ memory CD8 T cells secreting granzyme B and IFN-g in response to different cytokine conditions. Each dot represents an individual donor, and data are pooled from five separate donors. Error bars represent mean ± SD. Statistical significance was determined by one-way- ANOVA with Tukey's multiple comparisons. * indicates $p<0.05$, ** indicates $p<0.01$, *** indicates $p<0.001$, and **** indicates $p<0.0001$.

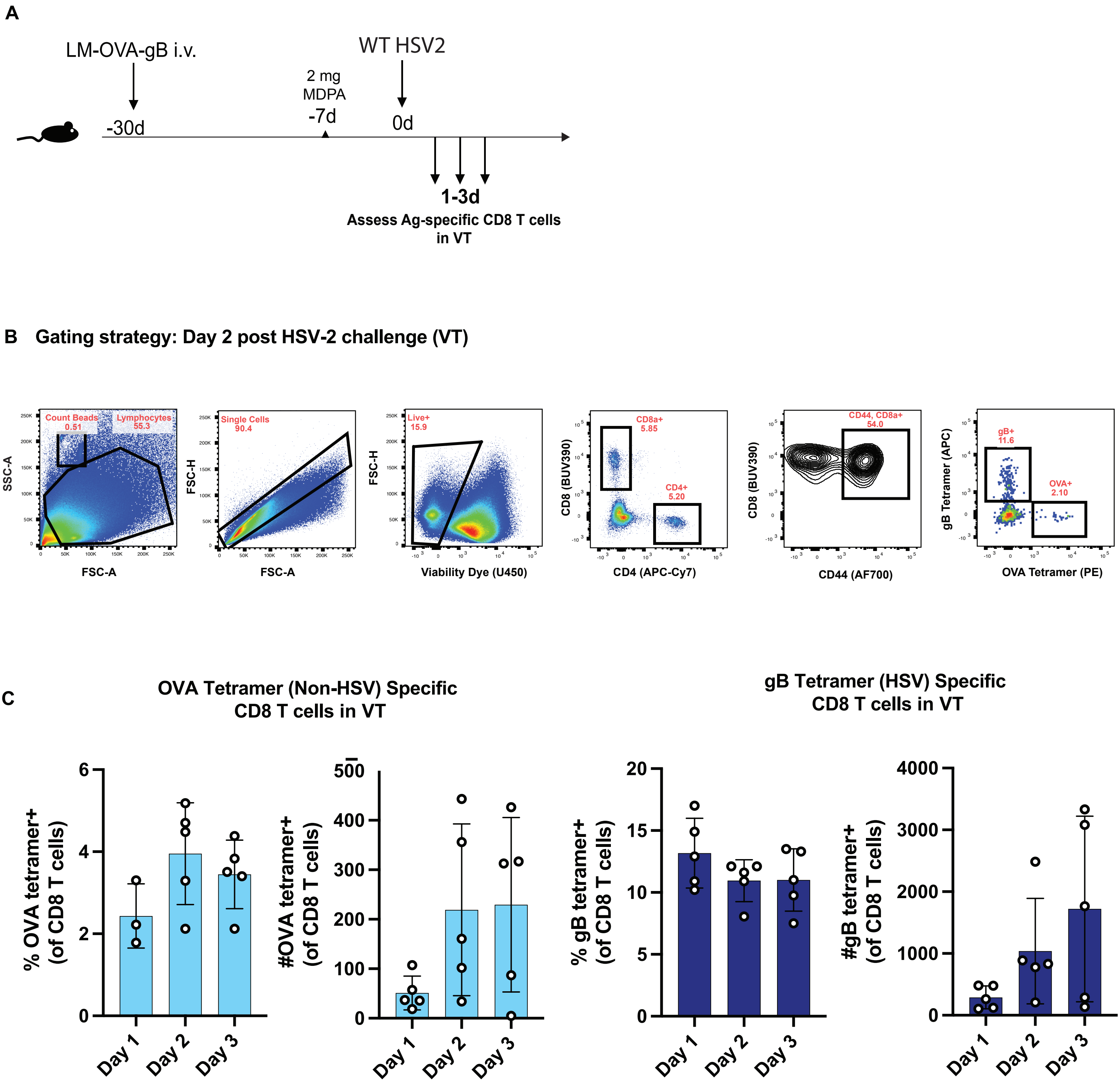

Supplemental Figure 1

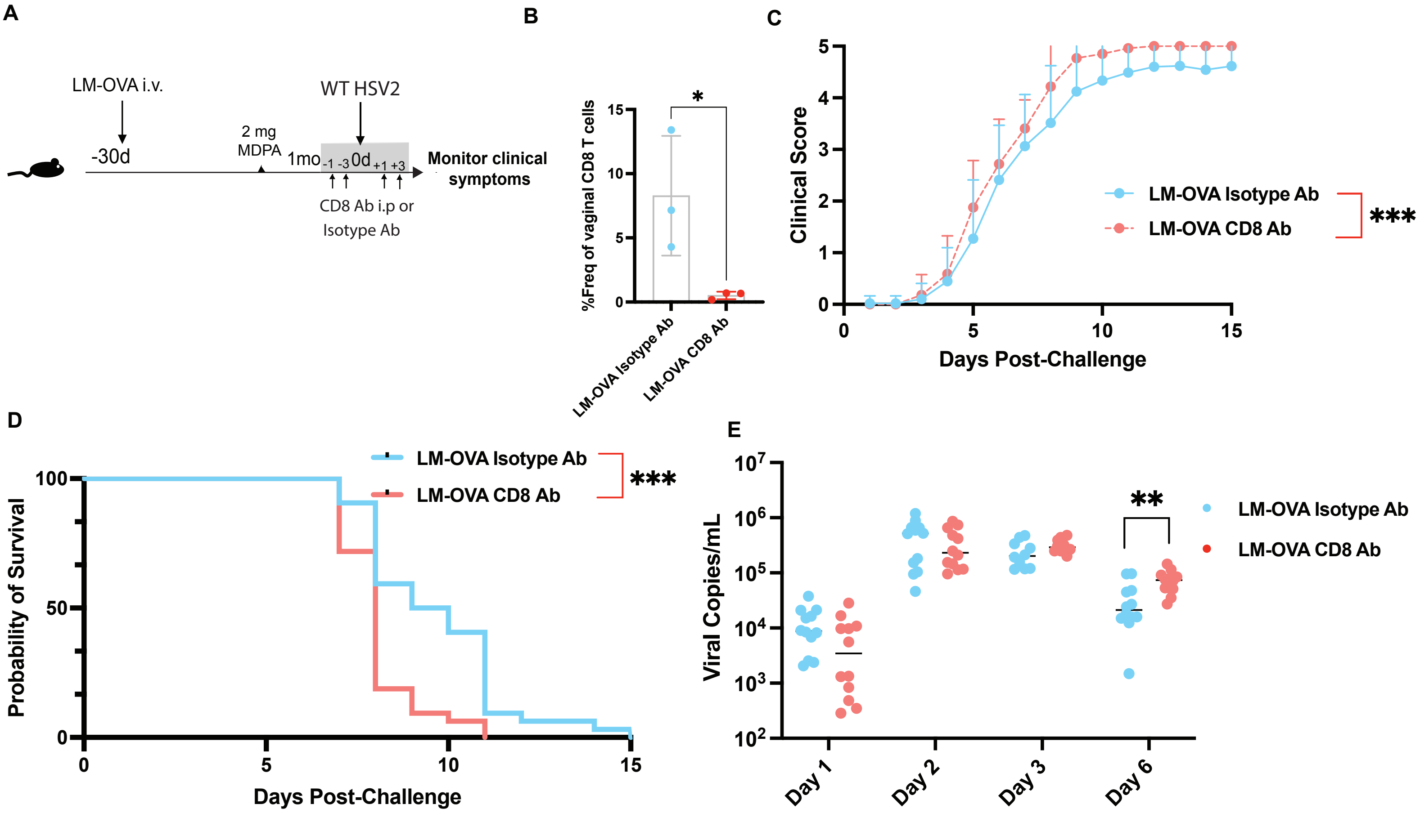

Supplemental Figure 2

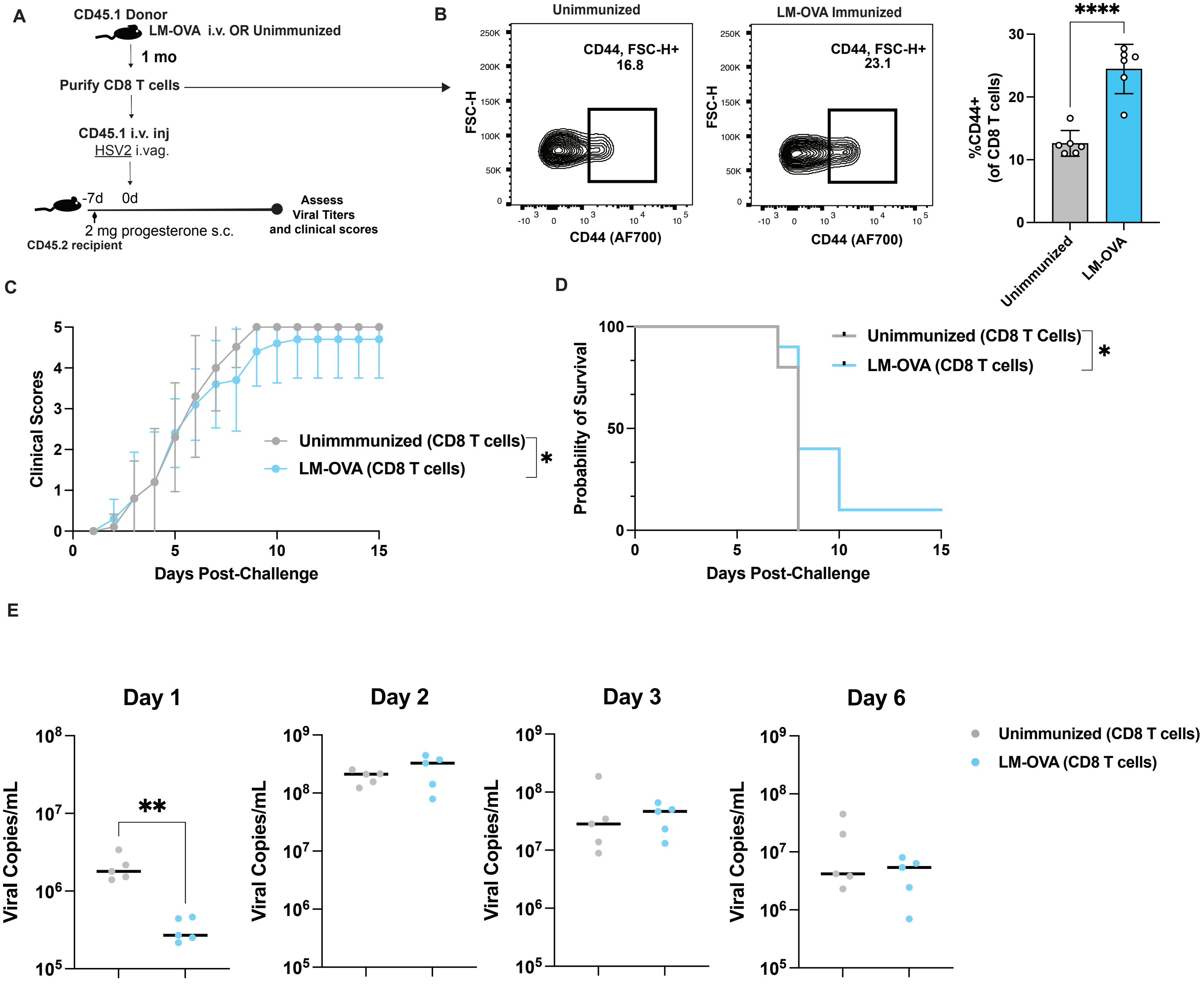

Supplemental Figure 3

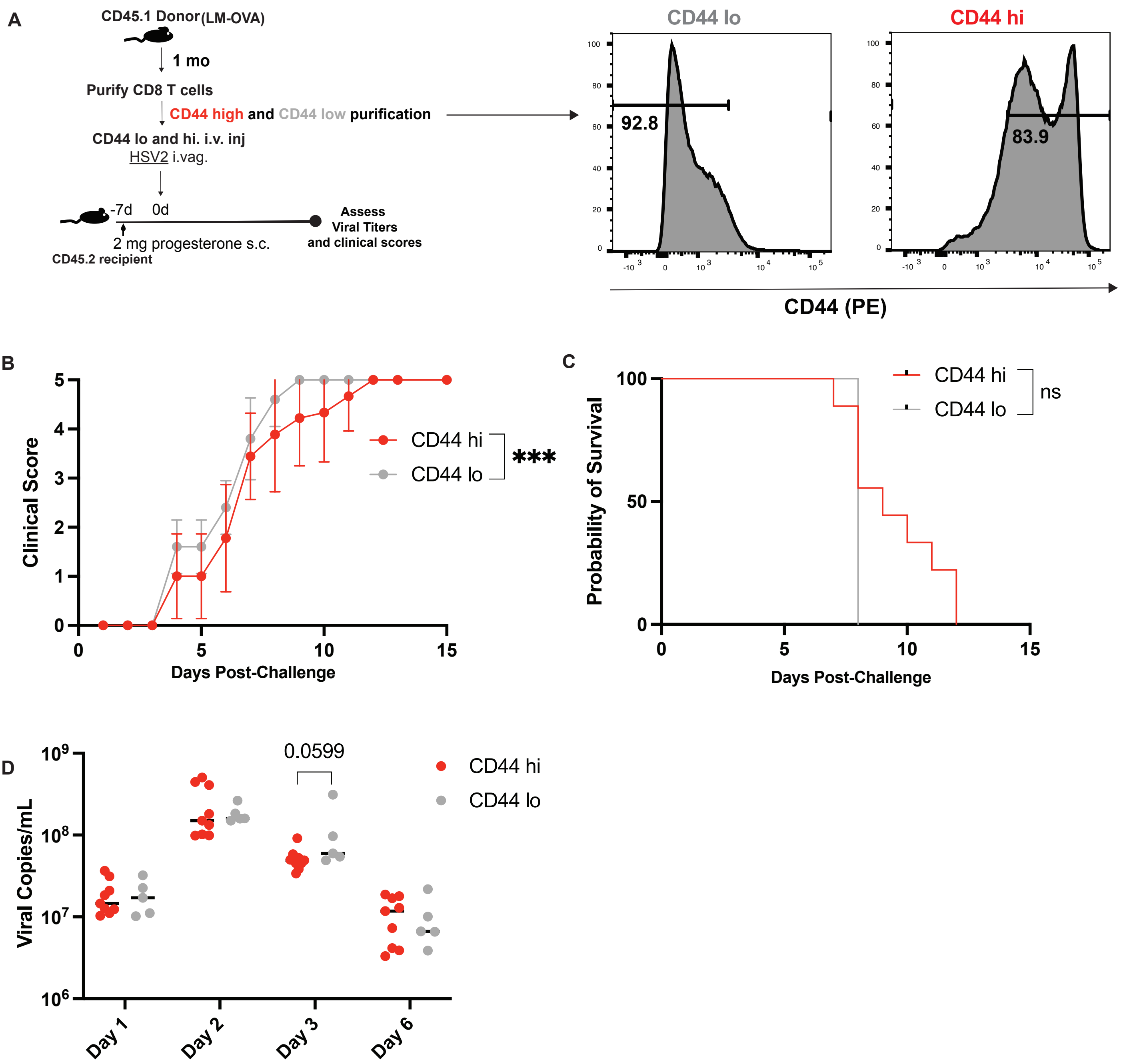

Supplemental Figure 4

A OVA tetramer+ CD44+ Population in Spleen

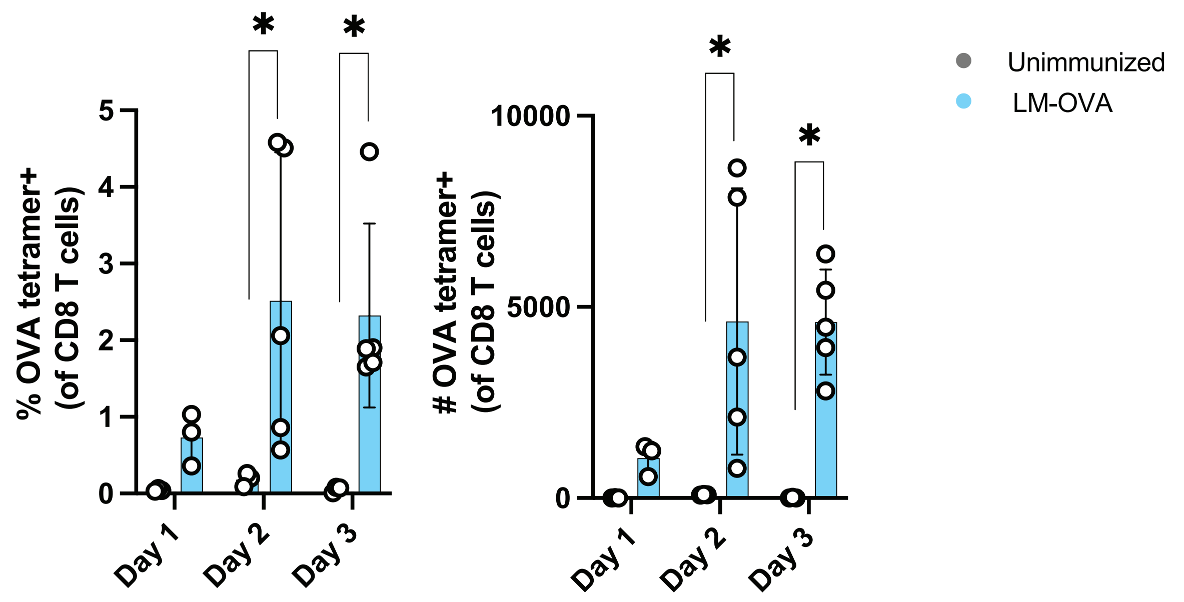

B OVA tetramer+ CD44+ Population in dLN

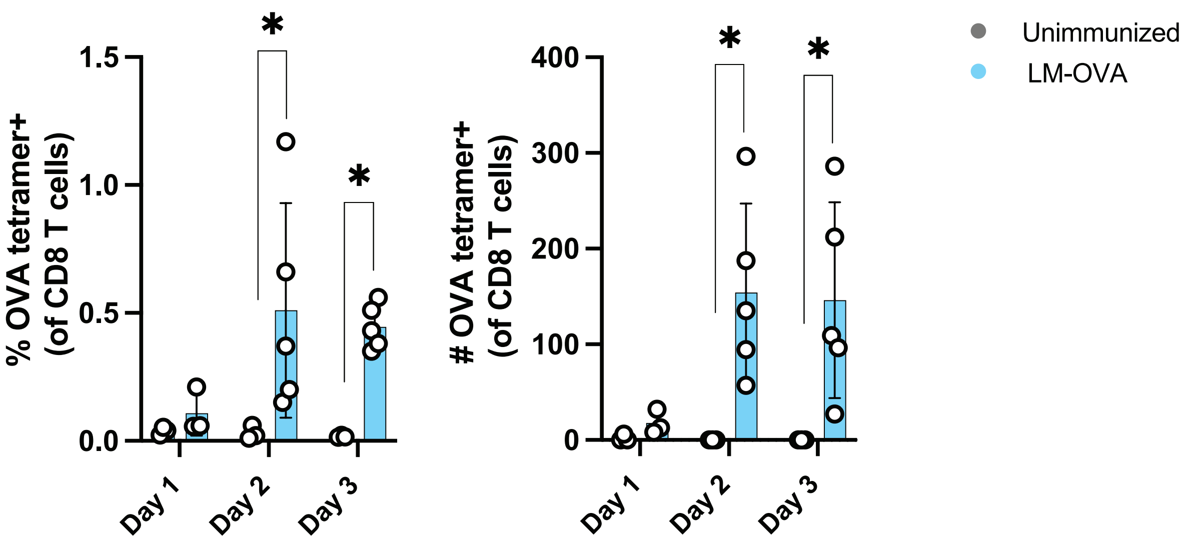

Supplemental Figure 5

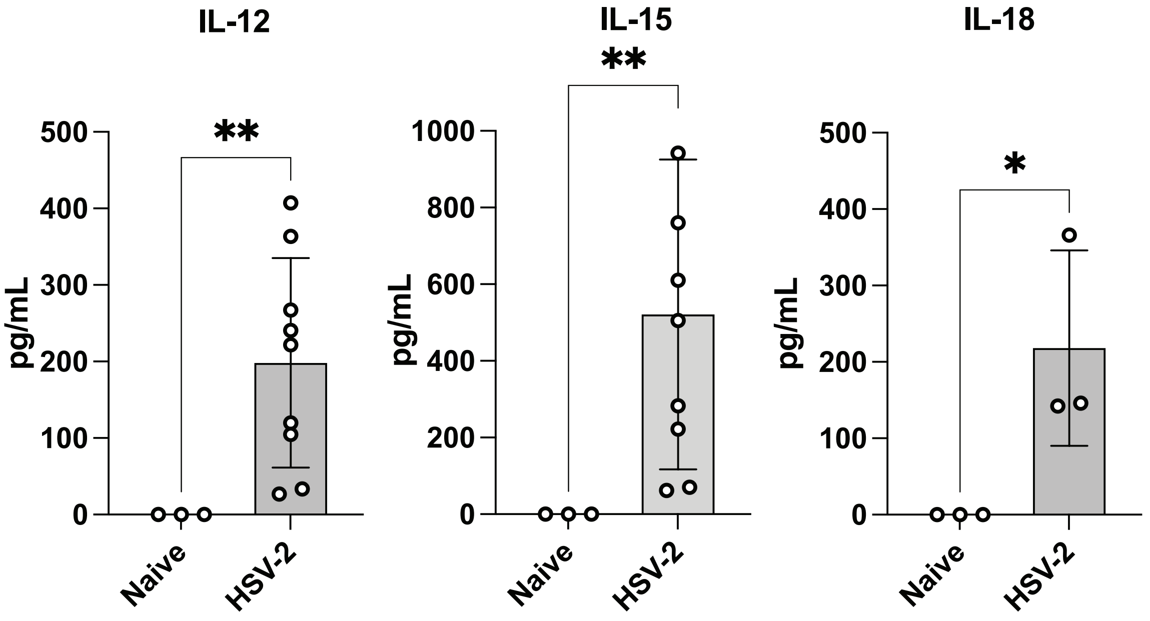

Supplemental Figure 6

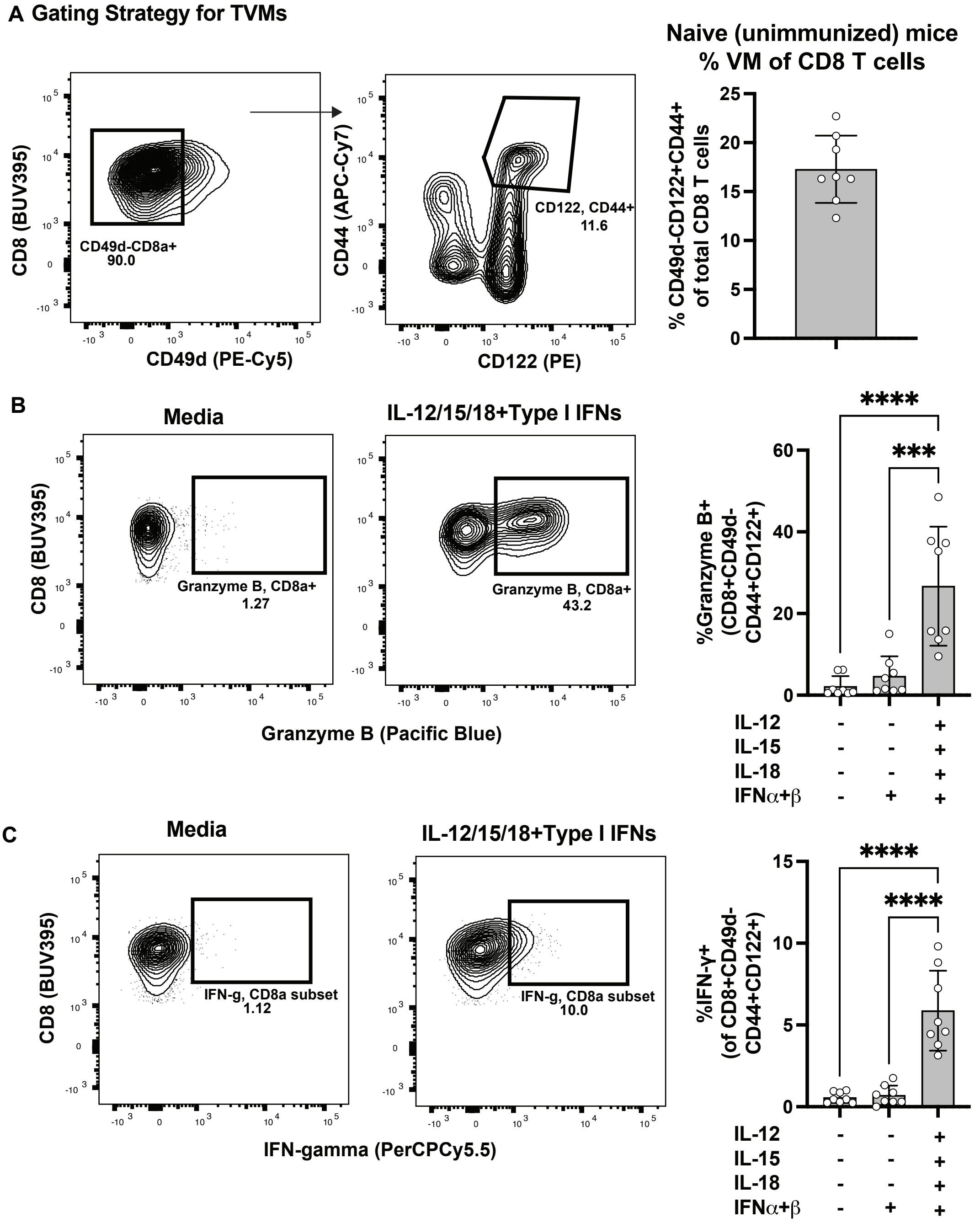

Supplemental Figure 7

Gating Strategy:Vaginal Tissue

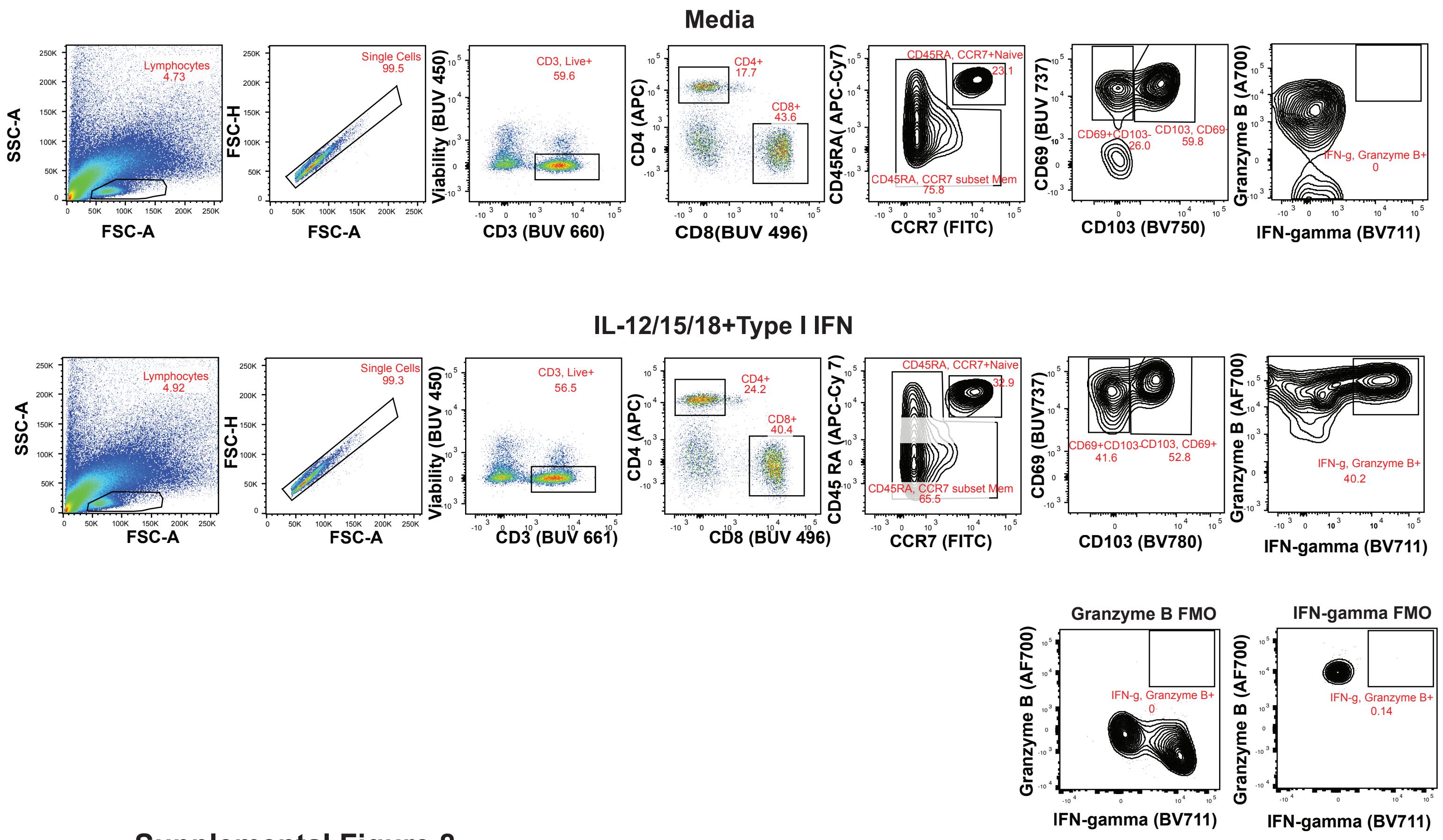

Supplemental Figure 8

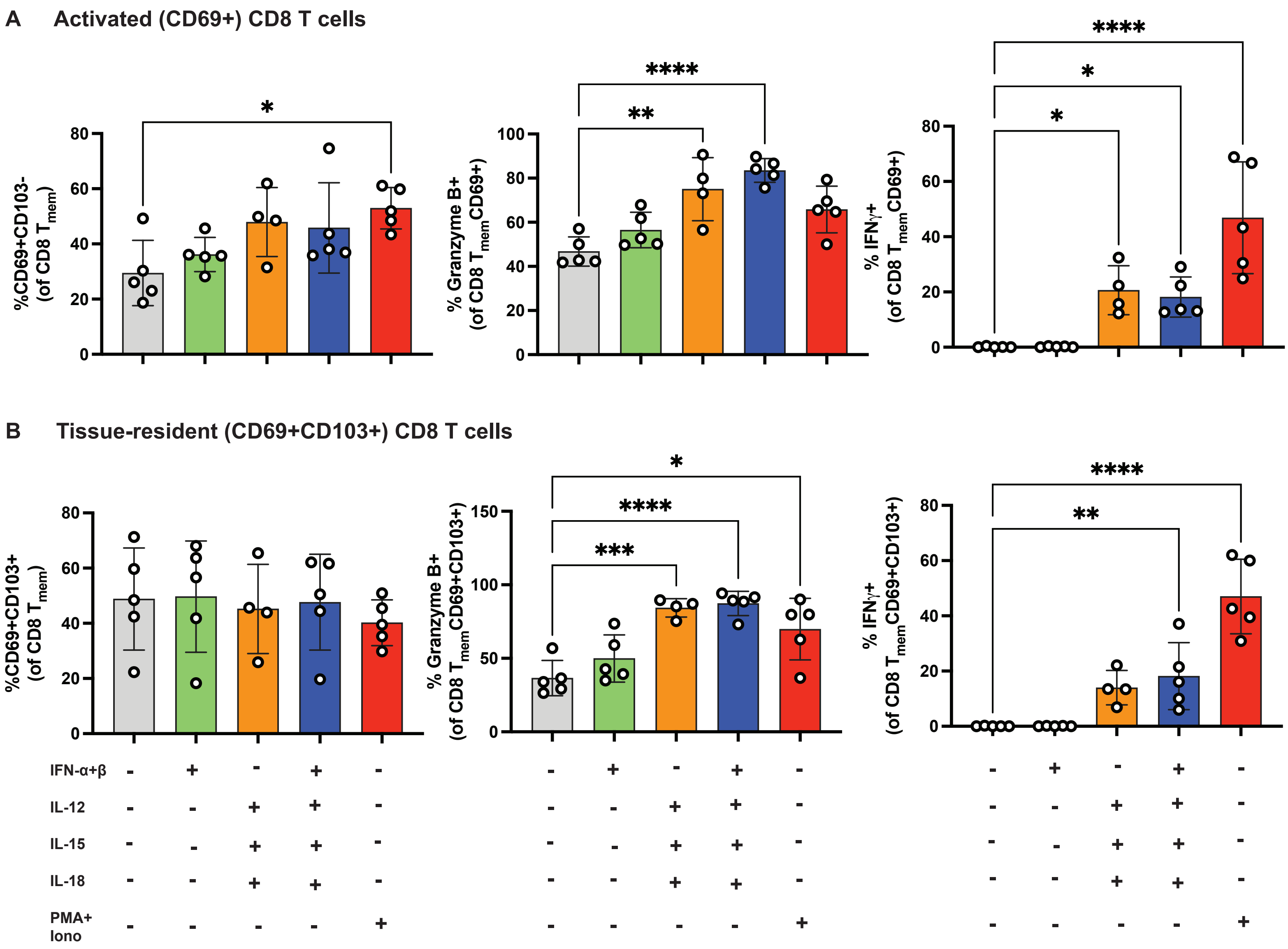

Supplemental Figure 9

Supplementary Table 1. Collaborative Cross Strains

Strain	Male	Female
CC001/Unc	2	2
CC002/Unc	2	2
CC003/Unc	1	0
CC004/TauUnc	2	0
CC005/TauUnc	2	4
CC007/Unc	2	2
CC008/GeniUncJ	1	2
CC009/Unc	2	2
CC010/GeniUncJ	2	2
CC011/UncJ	2	2
CC012/GeniUncJ	2	2
CC013/GeniUncJ	2	2
CC015/UncJ	2	2
CC017/UncJ	2	2
CC018/UncJ	2	1
CC021/Unc	2	2
CC024/GeniUncJ	2	2
CC025/GeniUncJ	2	0
CC026/GeniUncJ	2	2
CC027/GeniUncJ	2	2
CC028/GeniUncJ	2	2
CC029/UncJ	2	2
CC030/GeniUncJ	2	2
CC031/GeniUncJ	2	2
CC032/GeniUncJ	2	2
CC033/GeniUncJ	2	2
CC035/UncJ	2	2
CC036/UncJ	2	2
CC037/TauUnc	2	2
CC038/GeniUnc	2	2
CC039/Unc	2	2
CC040/TauUnc	2	2
CC041/TauUnc	2	2
CC042/GeniUnc	2	2
CC043/GeniUncJ	2	2
CC044/UncJ	2	2
CC045/GeniUncJ	4	2
CC046/Unc	2	2
CC049/TauUnc	2	2
CC051/TauUnc	2	2
CC053/Unc	2	2
CC057/UncJ	2	2
CC058/UncJ	4	2
CC059/TauUnc	2	2
CC060/UncJ	2	2
CC061/GeniUncJ	2	2
CC062/Unc	2	2
CC062/Unc	2	2

CC068/TauUncJ	2	2
CC071/TauUnc	2	2
CC072/TauUnc	2	2
CC074/UncJ	2	2
CC075/UncJ	2	2
CC078/UncJ	2	2
CC079/TauUncJ	2	2
CC080/TauUncJ	2	2
CC081/Unc	2	2
CC083/UncJ	2	2
CC084/TauUncJ	2	2

Supplementary Table 2. Murine bystander function panel for phenotyping

Conjugate	Antigen	Clone	Vendor	Dilution	Catalog
BV510	LIVE/DEAD Aqua Fixable Viability Dye (AViD)	N/A	Thermo Fisher Scientific	1: 500	L34957
	CD16/32 (Fc Block)	24.G2	BD Biosciences	1: 300	553141
Diluted in PBS 30-minute incubation at 4^0C					
BUV737	CD62L	MEL14		1:200	740218
BUV395	CD8α	53-6.7	BD Biosciences	1:300	563786
PE-Cy7	CD69	H1.2F3	Thermo Fisher Scientific	1:100	25-0691-82
APC-e780	CD44	IM7	Thermo Fisher Scientific	1:300	47-0441-82
AF700	CD4	RM4-5	Thermo Fisher Scientific	1:800	56-0042-82
BV650	CD45	30-F11	Biolegend	1:800	123149
BV710	NKG2D	CX5	BD biosciences	1:200	563688
PerCPCy5.5	Nur77	12.14	Thermo Fisher Scientific	1:200	46-5965-82
PE(Premium Grade, S21388, ThermoFisher)	Ova-Tetramer		Fred Hutch immune monitoring Core	1:100	
APC-Streptavidin(Premium Grade, S32362, ThermoFisher)	gB Tetramer		Fred Hutch immune monitoring Core	1:130	
Fixation in 1x eBioscience FOXP3 fixation/permeabilization buffer					
Pacific Blue<br>BV605	Granzyme B<br>Ki67	GB11<br>16A8	Biolegend<br>Biolegend	1:100<br>1:400	515408<br>652413

Supplementary Table 3. Murine bystander function panel for phenotyping and bystander-activating cytokine stimulations

Conjugate	Antigen	Clone	Vendor	Dilution	Catalog
BV510	LIVE/DEAD Aqua Fixable Viability Dye (AViD)	N/A	Thermo Fisher Scientific	1: 500	L34957
	CD16/32 (Fc Block)	24.G2	BD Biosciences	1: 300	553141
Diluted in PBS 30-minute incubation at 4^0C					
BUV737	CD62L	MEL14	BD Biosciences	1:200	740218
BUV395	CD8α	53-6.7	BD Biosciences	1:300	563786
PE-Cy7	CD69	H1.2F3	Thermo Fisher Scientific	1:100	25-0691-82
APC-e780	CD44	IM7	Thermo Fisher Scientific	1:300	47-0441-82
PE-CF594	CD49d	5H4	BD biosciences	1:800	564395
PE	CD122	5H4	Thermo Fisher Scientific	1:400	12-1221-82
AF700	CD4	RM4-5	Thermo Fisher Scientific	1:800	56-0042-82
BV650	CD45	30-F11	Biolegend	1:800	123149
Fixation in 1x eBioscience FOXP3 fixation/permeabilization buffer					
PerCP-Cy5.5	IFNγ	XMG 1.2	Biolegend	1:1000	505822
Pacific Blue	Granzyme B	GB11	Biolegend	1:100	515408

Supplementary Table 4. Human bystander function panel for bystander-activating cytokine stimulations

Conjugate	Antigen	Clone	Vendor	Dilution	Catalog
BUV450	Viability		Thermo Fisher Scientific	1:1000	L23105
	Diluted in PBS 30-minute incubation at 4^0C				
BUV496	CD8	RPA-T8	BD Biosciences	1:500	612943
BUV661	CD3	UCHT1	BD Biosciences	1:200	565065
BUV737	CD69	FN50	BD Biosciences	1:50	564439
BUV805	CD45	HI30	BD Biosciences	1:200	564914
BV750	CD103	Ber-ACT8	BD Biosciences	1:250	747099
AF488	CCR7	G043H7	Biolegend	1:83	353205
APC-H7	CD45RA	HI100	B BD Biosciences	1:40	560674
	Fixation in 1x eBioscience FOXP3 fixation/permeabilization buffer				
BV711	IFN-γ	4S.B3	Biolegend	1:40	502531
AF700	GranzymeB	GB11	BD Biosciences	1:83.3	560213
